# Supplementary material for: Prevalence and associated risk factors of intestinal parasitic infections among children in pastoralist and agro-pastoralist communities in the Adadle woreda of the Somali Regional State of Ethiopia
Source: PLoS Negl Trop Dis. 2023 Jul 3;17(7):e0011448. doi: 10.1371/journal.pntd.0011448 (PMC10348586; doi:10.1371/journal.pntd.0011448)
Supplement: S1 Fig — (DOCX) [file pntd.0011448.s001.docx]

**Screening**

Kebeles: N = 8

Children: N = 583

**Sampling**

Total: N = 366

Parasitology: N = 366

Questionnaire: N = 350

Not included: N = 212

Due to sample size

completion or withdrawal

**Analysis**

Perfect match: N = 345 Complete parasitology

and questionnaire

Partially missing:

Parasitology missing: N = 5

Questionnaire missing: N = 21

Parasitology prevalence: N = 366

Household level analyses: N = 358

Uni & multivariate analyses: N = 345

**S1 Figure. Methodological and analytical flow of study.**
