# Supplementary material for: Prevalence and associated risk factors of intestinal parasitic infections among children in pastoralist and agro-pastoralist communities in the Adadle woreda of the Somali Regional State of Ethiopia
Source: PLoS Negl Trop Dis. 2023 Jul 3;17(7):e0011448. doi: 10.1371/journal.pntd.0011448 (PMC10348586; doi:10.1371/journal.pntd.0011448)
Supplement: S2 Fig — Alternative informational figure to Table 3, with Somali translations. (PDF) [file pntd.0011448.s002.pdf]

Where do you source your drinking water?

Biyaha La cabbi

Tanktruck, Borehole or Natural Spring

Biyaha booyada, Ceel, ama Biyaha Dur-durka

Overall: 7.5%
Pastoralist: 9.4%
Agropastoralist: 5.6%

Rainwater in birkads

Biyaha roobka

Overall: 49.4%
Pastoralist: 89.0%
Agropastoralist: 9.0%

River water

Biyaha wabiga

Overall: 43.0%
Pastoralist: 1.7%
Agropastoralist: 85.3%

Do you treat drinking water?

Daawaynta biyaha

Overall: 30.4%
Pastoralist: 14.9%
Agropastoralist: 46.3%

What type of toilet do you use?

Musqul

Pit latrine

Musqul

Overall: 3.9%
Pastoralist: 7.2%
Agropastoralist: 0.6%

Outdoors

Banaanka

Overall: 96.1%
Pastoralist: 92.8%
Agropastoralist: 99.4%

Do you share your toilet?

Musqusha lawadaago

Overall: 8.9%
Pastoralist: 5.5%
Agropastoralist: 12.4%

How do you dispose of waste?

Qashin qubka

Burn

Gubi

Overall: 15.9%
Pastoralist: 15.5%
Agropastoralist: 16.4%

Dump

Tuur

Overall: 84.1%
Pastoralist: 84.5%
Agropastoralist: 83.6%

How do you wash your child's hands?

Gacmaha dhaqidda ilmaha

Water only

Biyo kaliya

Overall: 92.3%
Pastoralist: 96.1%
Agropastoralist: 88.3%

Water and Soap

Biyo iyo Saabuun

Overall: 7.7%
Pastoralist: 3.9%
Agropastoralist: 11.7%

Household owns soap?

Qoysku saabuun bu haysta

Overall: 44.0%
Pastoralist: 40.9%
Agropastoralist: 47.2%

S2 Fig. Household WASH characteristics of agro-pastoralist and pastoralist children 2 - 5 years of age living in Adadle woreda, Somali region, Ethiopia. Data collected in the wet season 2021. Alternative figure to Table 3 in the manuscript. PA: Pastoralist; AP: Agro-pastoralist; Simple Somali translations are given under each question and answer, to make the figure understandable to multiple audiences. Overall N = 358; Pastoralist N = 181; Agro-pastoralist N = 177. Images were created using Biorender.com.
