# Supplementary material for: Prevalence and associated risk factors of intestinal parasitic infections among children in pastoralist and agro-pastoralist communities in the Adadle woreda of the Somali Regional State of Ethiopia
Source: PLoS Negl Trop Dis. 2023 Jul 3;17(7):e0011448. doi: 10.1371/journal.pntd.0011448 (PMC10348586; doi:10.1371/journal.pntd.0011448)
Supplement: S1 Table — (DOCX) [file pntd.0011448.s003.docx]

**S1 Table. Sociodemographic household characteristics of agro-pastoralist and pastoralist children aged 2 - 5 years living in Adadle woreda, Somali region, Ethiopia**.

| **Characteristic** | **Agro-pastoralist**  **N = 177** | **Pastoralist**  **N = 181** | **Overall**  **N = 358** |
| --- | --- | --- | --- |
| **Household language** |  |  |  |
| Somali | 177 (100.0%) | 181 (100.0%) | 358 (100.0%) |
| **Household religion** |  |  |  |
| Islam | 177 (100.0%) | 181 (100.0%) | 358 (100.0%) |
| **Head of household is literate** | 47 (26.6%) | 45 (24.9%) | 92 (25.7%) |
| **Mother of child is literate** | 45 (25.4%) | 41 (22.7%) | 86 (24.0%) |
| **Household has a mobile phone** | 110 (62.1%) | 113 (62.4%) | 223 (62.3%) |
| Data are presented as n (%). | | | |
