# Supplementary material for: Prevalence and associated risk factors of intestinal parasitic infections among children in pastoralist and agro-pastoralist communities in the Adadle woreda of the Somali Regional State of Ethiopia
Source: PLoS Negl Trop Dis. 2023 Jul 3;17(7):e0011448. doi: 10.1371/journal.pntd.0011448 (PMC10348586; doi:10.1371/journal.pntd.0011448)
Supplement: S2 Table — (DOCX) [file pntd.0011448.s004.docx]

**S2 Table.** **Household animal herds of agro-pastoralist and pastoralist children aged 2 - 5 years of age living in Adadle woreda, Somali region, Ethiopia**.

| **Characteristic** | **Agro-Pastoralist, N = 177** | **Pastoralist, N = 181** | **Overall, N = 358** |
| --- | --- | --- | --- |
| **Household herd size** | 26 (13.0, 39) | 24 (6.0, 38) | 25 (9.0, 39) |
| **Animals kept inside house** | 102 (57.6%) | 142 (78.5%) | 244 (68.2%) |
| **Number of cattle** |  |  |  |
| No | 9 (5.1%) | 126 (69.6%) | 135 (37.7%) |
| 1-5 cattle | 88 (49.7%) | 47 (26.0%) | 135 (37.7%) |
| 6+ cattle | 80 (45.2%) | 8 (4.4%) | 88 (24.6%) |
| **Number of camels** |  |  |  |
| 0 | 162 (91.5%) | 115 (63.5%) | 277 (77.4%) |
| 1-3 | 10 (5.6%) | 34 (18.8%) | 44 (12.3%) |
| 4+ | 5 (2.8%) | 32 (17.7%) | 37 (10.3%) |
| **Number of goats** |  |  |  |
| 0 | 65 (36.7%) | 22 (12.2%) | 87 (24.3%) |
| 1-15 | 68 (38.4%) | 75 (41.4%) | 143 (39.9%) |
| 16+ | 44 (24.9%) | 84 (46.4%) | 128 (35.8%) |
| **Number of sheep** |  |  |  |
| 0 | 80 (45.2%) | 101 (55.8%) | 181 (50.6%) |
| 1-10 | 50 (28.2%) | 56 (30.9%) | 106 (29.6%) |
| 11+ | 47 (26.6%) | 24 (13.3%) | 71 (19.8%) |
| **Number of donkeys** |  |  |  |
| 0 | 34 (19.2%) | 77 (42.5%) | 111 (31.0%) |
| 1 | 72 (40.7%) | 74 (40.9%) | 146 (40.8%) |
| 2+ | 71 (40.1%) | 30 (16.6%) | 101 (28.2%) |
| **Number of chickens** |  |  |  |
| 0 | 159 (89.8%) | 181 (100.0%) | 340 (95.0%) |
| 1-5 | 10 (5.6%) | 0 (0.0%) | 10 (2.8%) |
| 6+ | 8 (4.5%) | 0 (0.0%) | 8 (2.2%) |
| Data are presented as median (IQR) or n (%) unless otherwise stated. | | | |
