# Supplementary material for: Prevalence and associated risk factors of intestinal parasitic infections among children in pastoralist and agro-pastoralist communities in the Adadle woreda of the Somali Regional State of Ethiopia
Source: PLoS Negl Trop Dis. 2023 Jul 3;17(7):e0011448. doi: 10.1371/journal.pntd.0011448 (PMC10348586; doi:10.1371/journal.pntd.0011448)
Supplement: S1 File — (DOCX) [file pntd.0011448.s005.docx]

**STROBE Checklist** — checklist of items that should be included in reports of observational studies – Cross-sectional study

**Completed for current study:** Prevalence and associated risk factors of intestinal parasitic infections in young pastoralist and agro-pastoralist children in the Somali Region of Ethiopia

|  | | Item No. | | | | Recommendation | | Page  No. | Relevant text from manuscript | |
| --- | --- | --- | --- | --- | --- | --- | --- | --- | --- | --- |
| **Title and abstract** | | 1 | | | | (*a*) Indicate the study’s design with a commonly used term in the title or the abstract | | 2 | - “We assessed the prevalence of IPIs and associated risk factors during the wet season from May-June 2021 in 366 children aged 2 to 5 years in four agro-pastoralist and four pastoralist *kebeles* (wards) in Adadle *woreda* (district) of the Shebelle zone, ESRS.” | |
|  |  |  |  |  |  | (*b*) Provide in the abstract an informative and balanced summary of what was done and what was found | | 2 | - **“Methodology:** We assessed the prevalence of IPIs and associated risk factors during the wet season from May-June 2021 in 366 children aged 2 to 5 years in four agro-pastoralist and four pastoralist *kebeles* (wards) in Adadle *woreda* (district) of the Shebelle zone, ESRS. Household information, anthropometric measurements, and stool samples were obtained from included children. Parasites were identified microscopically using Kato-Katz and direct smear methods. Risk factors were assessed using general estimating equation models accounting for clustering.” - **“Principal Findings:** Overall prevalence of IPIs was 35%: 30.6% for single infections and 4.4% for poly-parasitic infections. Intestinal protozoan prevalence was 24.9%: 21.9% *Giardia intestinalis*, and 3.0% *Entamoeba* spp.. Intestinal helminth prevalence was 14.5%: 12.8% *Ascaris lumbricoides*, 1.4% hookworm (*Ancylostoma duodenale* /*Necator americanus*.), and 0.3% *Hymenolepis nana*. *G. intestinalis* infection was associated with drinking water sourced from the river (aOR 15.6, 95%CI 6.84, 35.4) and from collected rainwater (aOR 9.48, 95%CI 3.39, 26.5), with toilet sharing (aOR 2.93, 95%CI 1.36, 6.31) and with household ownership of cattle (1-5 cattle: aOR 1.65, 95%CI 1.13, 2.41; 6+ cattle: aOR 2.07, 95%CI 1.33, 3.21) and chickens (aOR 3.80, 95%CI 1.77, 8.17). *A. lumbricoides* infection was associated with children 36 to 47 months old (aOR 1.92, 95%CI 1.03, 3.58).” | |
| Introduction | | | | | | | | |  | |
| Background/rationale | | 2 | | | | Explain the scientific background and rationale for the investigation being reported | | 3 - 4 | - “Intestinal parasitic infections (IPIs) remain one of the most common infectious diseases.” - “Children are especially at risk for IPIs.” - “In Ethiopia, IPI prevalence has been found to be between 42% and 53% in children…” - “There is no data on prevalence in the wet season nor information on associated risk factors of IPIs, leaving a knowledge gap regarding IPIs in pastoralist communities in the ESRS.” | |
| Objectives | | 3 | | | | State specific objectives, including any prespecified hypotheses | | 4 - 5 | - “As pastoralists live in close connection and proximity to one another, their animals, and their environment year-round, it is expected that the sharing of microbial species, including intestinal parasites, is high. This study assessed the prevalence of intestinal parasitic infections and associated risk factors in children aged 2 to 5 years in pastoralist and agro-pastoralist communities in the Adadle *woreda* (district) of the Shabelle zone of the Ethiopian Somali Regional State (ESRS)”. | |
| Methods | | | | | | | | |  | |
| Study design | | 4 | | | | Present key elements of study design early in the paper | | 6 | - “The study was a cross-sectional study in the Adadle *woreda* (district) in the Shabelle zone of the Somali Regional State of Ethiopia and was carried out from May 2021 until June 2021 during the wet season, known locally as *gu-‘ga*.” - “children aged 2 - 5 years” | |
| Setting | | 5 | | | | Describe the setting, locations, and relevant dates, including periods of recruitment, exposure, follow-up, and data collection | | 6 - 8 | - “The study is a cross-sectional study in the Adadle *woreda* (district) in the Shabelle zone of the Somali Regional State of Ethiopia and was carried out from May 2021 until June 2021 during the wet season, known locally as *gu-‘ga*. Adadle is located 17 km from the city of Gode in the lowlands of the Wabi Shabelle River subbasin and experiences a mean annual temperature of 32˚C and a mean annual rainfall of 300 mm. The altitude is 300-500 meters above sea-level and 80% of the land is flat, while 20% is undulated. The pastoralist and agro-pastoralist communities in ESRS and Adadle rely mainly on their animals for food and livelihood and live largely outside of modern systems.” - “Stakeholders (community leaders and health officials) in Adadle were approached at each level (district, ward, sub-ward) and invited to help determine which kebeles in Adadle could be included in the present study.” - “All children were screened for stunting (height for age) and wasting (mid-upper arm circumference for height; MUAC), according to the 2006 WHO growth standards [1].” - “A detailed questionnaire using the Open Data Kit (ODK) software [2] was administered in the local language by field workers to the mother of the child.” | |
| Participants | | 6 | | | | *Cross-sectional study*—Give the eligibility criteria, and the sources and methods of selection of participants | | 6 - 7 | - “Stakeholders (community leaders and health officials) in Adadle were approached at each level (district, ward, sub-ward) and invited to help determine which kebeles in Adadle could be included in the present study. From the 15 kebeles in Adadle, four pastoralist (Malkasalah, Todob, Harsug, Kulmis) and four agro-pastoralist (Bursaredo, Gabal, Higlo, Boholhagare) kebeles were randomly selected (**Fig 1**).” - “A pre-enrolment screening was carried out at the local health centre in each kebele, for all children aged 2 - 5 years.” - “All children were screened for stunting (height for age) and wasting (mid-upper arm circumference for height; MUAC), according to the 2006 WHO growth standards [1]. All screened children who were severely wasted based on their weight for height z-score (WHZ < -3) or severely stunted based on their height for age z-score (HAZ < -3) were automatically included in the study. Screened children not presenting as severely stunted and/or severely wasted were randomly selected (random number generation) from an excel file containing all pre-screened households/children in the community, until the maximum sample size was reached for each kebele and the overall study.” - **Exclusion criteria:** “Children were excluded if they were older than 5 years, younger than 2 years and/or had taken antibiotics in the last 14 days.” | |
| Variables | | 7 | | | | Clearly define all outcomes, exposures, predictors, potential confounders, and effect modifiers. Give diagnostic criteria, if applicable | | 8 - 9 | - “Stunting, wasting, and underweight were defined as HAZ, WHZ, WAZ < -2, while acute malnutrition (low MUAC) was defined by MUAC < 12.5 cm.” - “(1) child anthropometric measures, current health and health history of the child (2) household characteristics and assets, and type and number of household animals, (3) WASH behaviours of child and household, (4) a nutritional survey of the child, and finally (5) breastfeeding and birth history of mother-child.” - “One portion was used for the quantification of helminth ova using the thick-smear Kato-Katz detection method [3] under a microscope at 10x and 40x magnification, which allows for the detection of common helminth species such as *Ancylostoma* spp. */Necator* spp. (hookworm), *Hymenolepis nana*, *Trichuris trichiura, Ascaris lumbricoides*, and *Schistosoma mansoni*. Another two portions were used for the detection of protozoan cysts and trophozoites, such as *Giardia intestinalis* and *Entamoeba* spp. using the direct smear method in duplicate.” | |
| Data sources/ measurement | | 8* | | | | For each variable of interest, give sources of data and details of methods of assessment (measurement). Describe comparability of assessment methods if there is more than one group | | 7 - 9 | - “Anthropometric measurements of height, weight, and mid-upper arm circumference (MUAC) were measured.” - “A detailed questionnaire using the Open Data Kit (ODK) software [2] was administered in the local language by field workers to the mother of the child. The questionnaire was divided into several sections: (1) child anthropometric measures, current health and health history of child (2) household characteristics and assets, and type and number of household animals, (3) WASH behaviours of child and household, (4) a nutritional survey of the child, and finally (5) breastfeeding and birth history of mother-child.” - “Stool samples were collected by the mothers in sterile specimen cups after detailed instruction by a trained field worker in the local language. The samples were aliquoted and then immediately transferred to a cold box containing ice. All parasitology analyses were performed in the field in each kebele health clinic by trained parasitologists from Jigjiga University.” | |
| Bias | | 9 | | | | Describe any efforts to address potential sources of bias | | 7 - 8 | - “From the included kebeles, four pastoralist (Malkasalah, Todob, Harsug, Kulmis) and four agro-pastoralist (Bursaredo, Gabal, Higlo, Boholhagare) kebeles were randomly selected” - “Screened children not presenting as severely stunted and/or severely wasted were randomly selected (random number generation) from an excel file containing all pre-screened households/children in the community, until the maximum sample size was reached for each kebele and the overall study.” - “Birth records often do not exist in pastoralist communities; therefore, age in months was estimated based on group discussions with the child’s mother and other familial and community members to determine seasonal (floods, month of year, drought) and festive events (Ramadan, other religious events) that occurred before or after the birth of the child.” | |
| Study size | | 10 | | | | Explain how the study size was arrived at | | 6 | - “The sample size was determined based on several components, as this study was part of a larger project studying antimicrobial resistance and the microbiome from a One Health perspective. A previous study in children aged 2 to 5 years in this region showed a prevalence of intestinal parasitic infections of 42% [4]. As we expected clustering on the *kebele* (ward) level, the intra-cluster correlation coefficient was assumed to be 0.15. We chose a 95% confidence interval, power of 80%, alpha of 0.05, and a margin of error of 10%. Based on these factors and to achieve the necessary sample size for all aims of the larger research project, we calculated a sample size of 360 eligible children, 180 each in pastoralist and agro-pastoralist communities.” | |
| Quantitative variables | | 11 | | | | Explain how quantitative variables were handled in the analyses. If applicable, describe which groupings were chosen and why | | 9 - 10 | - “All statistical analyses were performed in R Statistical software version 4.0.4 [5]. Tables were generated using the gtsummary package [6].” - “Individual observations where the mother answered "don’t know" are listed as “Unknown" in descriptive tables. Categorization of numerical variables, such as ownership of cattle, were based on the median value as a cut-off point. Univariable and multivariable analysis were carried out using the general estimating equation logistic regression model for binary outcomes using the geepack package [7], to account for clustering on the kebele level. Variables in the multivariable model were pre-selected based on the published literature and complemented with variables associated in the univariable analysis (p < 0.2) [8].” | |
| Statistical methods | | 12 | | | | (a) Describe all statistical methods, including those used to control for confounding | | 10 | - “Briefly, the ODK questionnaire data (including anthropometric data) and parasitology data were merged according to each child’s identification number. We used the available case population for all analyses. For household-level metrics, missing data was addressed by applying the completed questionnaires of siblings in the same household. Missing data for individual metrics (such as anthropometric metrics) were not able to be recovered and these observations were therefore left out of analyses.” | |
|  | |  | | | | (b) Describe any methods used to examine subgroups and interactions | | 9 - 10 | - “Univariable and multivariable analysis were carried out using the general estimating equation logistic regression model for binary outcomes using the geepack package [7], to account for clustering on the kebele level.” | |
|  | |  | | | | (c) Explain how missing data were addressed | | 9 - 10 | - “We used the available case population for all analyses. For household-level metrics, missing data was addressed by applying the completed questionnaires of siblings in the same household. Missing data for individual metrics (such as anthropometric metrics) were not able to be recovered and these observations were therefore left out of analyses. Individual observations where the mother answered "don’t know" are listed as “Unknown" in descriptive tables.” | |
|  | |  | | | | (d) Cross-sectional study—If applicable, describe analytical methods taking account of sampling strategy | | 9 - 10 | - “Univariable and multivariable analysis were carried out using the general estimating equation logistic regression model for binary outcomes using the geepack package [7], to account for clustering on the kebele level. Variables in the multivariable model were pre-selected based on the published literature and complemented with variables associated in the univariable analysis (p < 0.2) [8].” | |
|  | |  | | | | (e) Describe any sensitivity analyses | | NA | - NA | |
| Results | | | | | | | | | | |
| Participants | 13* | | | (a) Report numbers of individuals at each stage of study—eg numbers potentially eligible, examined for eligibility, confirmed eligible, included in the study, completing follow-up, and analysed | | | 11 | | - “A total of 366 children aged between two to five years from 270 households spread between eight kebeles in the Adadle woreda (district), Shabelle zone of the Ethiopian Somali Regional State (ESRS) were included in the present study, of which 184 children were from pastoralist and 182 from agro-pastoralist communities. During the screening process, 7 children presented as severely stunted, 27 as severely wasted, and 3 as severely stunted and severely wasted, all of whom were included in the study. For all included children, parasitology reports were documented (N=366). However, for twenty-one children, a questionnaire was not recovered or completed, due to logistical issues with the software and field activities. Therefore, 345 children completed both a questionnaire and parasitology report (**S1 Fig**). By applying household-level data from completed questionnaires of siblings in the same household, 13 of the 21 children with a missing questionnaire were able to be recovered for the household-level descriptive analyses (N=358; **S1** **Fig**).” | |
|  |  |  |  | (b) Give reasons for non-participation at each stage | | | 7 | | - “Children were excluded if they were older than 5 years, younger than 2 years and/or had taken antibiotics in the last 14 days.” - See **S1 Fig** | |
|  |  |  |  | (c) Consider use of a flow diagram | | | 11 | | - See **S1 Fig** | |
| Descriptive data | 14* | | | (a) Give characteristics of study participants (eg demographic, clinical, social) and information on exposures and potential confounders | | | 11 - 18 | | - “Participant characteristics are summarized in **Table 1**. The age and sex of enrolled children were distributed evenly, including between pastoralist and agro-pastoralist communities.” - “Height, weight, and mid-upper arm circumference (MUAC) were similarly distributed between pastoralist and agro-pastoralist children (**Table 2**).” - “The included agro-pastoralist kebeles all reside along or near the Shabelle River (**Fig 1**).” - “The included pastoralist kebeles (1: Todob; 2: Kulmis; 3: Malkasalah; 4: Harsug) did not reside near any rivers, but did flank road systems, and were between 48 - 75 km from Gode town (**Fig 1**).” - “Household water, sanitation and hygiene (WASH) characteristics showed some differences between pastoralist and agro-pastoralist communities and are summarized in **Table 3**.” - “Household ownership of domestic animals, which includes in the present study cattle, camel, goats, sheep, donkeys, and chickens, varied in quantity between the agro-pastoralist and pastoralist communities (**Fig 2; S2 Table)**.” | |
|  |  |  |  | (b) Indicate number of participants with missing data for each variable of interest | | | 11 | | - “For all included children, parasitology reports were documented (N=366). However, for twenty-one children, a questionnaire was not recovered or completed, due to logistical issues with the software and field activities. Therefore, 345 children completed both a questionnaire and parasitology report (**S1 Fig**). By applying household-level data from completed questionnaires of siblings in the same household, 13 of the 21 children with a missing questionnaire were able to be recovered for the household-level descriptive analyses (N=358; **S1** **Fig**).” | |
|  |  |  |  | *Cross-sectional study—*Report numbers of outcome events or summary measures | | | 18 - 19 | | - “The overall prevalence of intestinal parasitic infections (IPIs) was 35%, with a prevalence of 30.6% for single parasitic infections and 4.4% for poly-parasitic infections (coinfection with protozoan and helminth species). The infection pattern is summarized in **Table 4**.” | |
| Main results | 16 | | | (*a*) Give unadjusted estimates and, if applicable, confounder-adjusted estimates and their precision (eg, 95% confidence interval). Make clear which confounders were adjusted for and why they were included | | | 19 - 22 | | - “In univariate analysis, *G. intestinalis* infection was associated with children aged 36-47 months, source of drinking water, ownership of cattle and ownership of chickens. In multivariate analysis, *G. intestinalis* infection was associated with water source, toilet sharing, ownership of cattle and ownership of chicken (**Table 5**). Sourcing drinking water from rainwater stored in birkads (open shallow wells/ditches) had an aOR of 9.48 [3.39, 26.5], p-value < 0.001, while sourcing from river water had an aOR of 15.6 [6.84, 35.4], p-value < 0.001, when compared to sourcing water from a spring, tank truck, or borehole. Toilet sharing had an aOR of 2.93 [1.36,6.31], p-value = 0.006. Household ownership of cattle revealed an aOR of 1.65 [1.13 2.41], p-value = 0.009, when owning 1 - 5 cattle, and an aOR of 2.07 [1.33, 3.21], p-value = 0.001, when owning more than 6 cattle, compared with owning no cattle. Similarly, owning chickens had an aOR of 3.8 [1.77, 8.17], p-value < 0.001. All further variables included in multivariate analysis were non-significant. Univariate and multivariate was not possible for *Entamoeba* spp., due to low positive numbers.” - “In univariate analysis, *Ascaris lumbricoides* was associated with children aged 36-47 months and with owning more than 6 cattle (**Table 6**). In multivariate analysis, *A. lumbricoides* infection remained associated with children aged 36-47 months (aOR 1.92 [1.03, 3.58], p-value = 0.040) compared to children aged 23-35 months. All further variables included in multivariate analysis were non-significant. Univariate and multivariate was not possible for the other helminth parasites due to low positive numbers.” | |
|  |  |  |  | (*b*) Report category boundaries when continuous variables were categorized | | | 19 | | - “Household ownership of cattle revealed an aOR of 1.65 [1.13 2.41], p-value = 0.009, when owning 1 - 5 cattle, and an aOR of 2.07 [1.33, 3.21], p-value = 0.001, when owning more than 6 cattle, compared with owning no cattle. Similarly, owning chickens had an aOR of 3.8 [1.77, 8.17], p-value < 0.001.” | |
|  |  |  |  | (*c*) If relevant, consider translating estimates of relative risk into absolute risk for a meaningful time period | | | NA | | - NA | |
|  |  |  |  | Report other analyses done—eg analyses of subgroups and interactions, and sensitivity analyses | | | NA | | - NA | |
| Discussion | | | | | | | | | | |
| Key results | | | 18 | | Summarise key results with reference to study objectives | | | | 22 - 26 | - “To our knowledge, this is the first study assessing the impact of livestock animal keeping and WASH characteristics on the risk of intestinal parasitic infections (IPIs) in children 2 - 5 years of age living in pastoralist and agro-pastoralist communities in the ESRS.” - “A recent study performed in the same district with pastoralist communities during the dry (drought) season showed slightly higher overall IPI prevalence (47%) than in our study (35%), although the prevalence of *G. intestinalis* and *A. lumbricoides* were comparable [4]. - “Among the intestinal parasites detected in this study, *Giardia intestinalis* infection was the highest and was found to be associated with source of drinking water, toilet sharing, ownership of cattle, and ownership of chicken.” - “Household sharing of the toilet area was found to be associated with *Giardia intestinalis* infection, indicating that this may be a potential transmission point between households.” - “Ownership of cattle was also found to be significantly associated with *Giardia intestinalis* infection.” - “Children aged 36-47 months old were associated with *A. lumbricoides* infection.” |
| Limitations | | | 19 | | Discuss limitations of the study, taking into account sources of potential bias or imprecision. Discuss both direction and magnitude of any potential bias | | | | 27 | - “There are several limiting factors in the present study due to logistical constraints. First, due to the methods used (inclusion and exclusion criterion) in selecting children for this study, it is possible that we overestimated wasting and stunting in the population as well as underestimated IPIs: children receiving antibiotics for any type of illness (which could include symptomatic IPIs) were excluded from the study. Further, only one sample was able to be obtained and analysed per child, due to logistical constraints, which may underestimate the true prevalence of IPIs, since the diagnostic techniques used lack perfect sensitivity for certain intestinal parasites [9,10]. Given the strong associations of water source, toilet sharing, and cattle and chicken ownership with *G. intestinalis* infection in this study, future studies should include environmental and animal parasitology when assessing parasite prevalence and risk factors in communities in which there is a high rate of contact between humans, animals and their environment. In addition, the One Health approach in parasitology and pathogenic microorganism research is applicable beyond the few parasites analysed in this study; indeed, there are many parasites and microorganisms of human and animal health importance that are transmitted among and between humans and animals through environmental channels (water, soil, food, plants, air) [11–16]. Transmission events between humans, animals, and their environment would best be addressed using genomic techniques [17], and supported with qualitative information to place the transmission events in context and develop appropriate interventions in the given context [18,19]. Taking these approaches would likely improve the health of humans and their animals, as well as reduce the loss of food and income to illness [11,20].” |
| Interpretation | | | 20 | | Give a cautious overall interpretation of results considering objectives, limitations, multiplicity of analyses, results from similar studies, and other relevant evidence | | | | 22 - 28 | - “To our knowledge, this is the first study assessing the impact of livestock animal keeping and WASH characteristics on the risk of intestinal parasitic infections (IPIs) in children 2 - 5 years of age living in pastoralist and agro-pastoralist communities in the ESRS. In addition, this study adds to the limited body of research on the health of pastoralist communities living in the ESRS and in the horn of Africa, the latter geographic zone encompassing an estimated 20-30 million humans living a pastoral lifestyle [21,22].” - “A recent study performed in the same district with pastoralist communities during the dry (drought) season showed slightly higher overall IPI prevalence (47%) than in our study (35%), although the prevalence of *G. intestinalis* and *A. lumbricoides* were comparable [4].” - The prevalence of IPIs in agro-pastoralist and pastoralist children (aged 2 – 5 years), especially of *G. intestinalis*, are of regional public health concern, given the immediate and long-term health impacts of these types of infections in children. Drinking water source, toilet sharing and household ownership of cattle and chickens were found to be important risk factors for *G. intestinalis* infection in these communities. Children aged 36-47 months were associated with *A. lumbricoides* infection. We recommend additions and improvements to water, sanitation, and hygiene (WASH) infrastructure for use by semi-mobile pastoralists, with attention given to the unique relationship pastoralists have with their animals and environment. Any intervention should be implemented in a transdisciplinary manner with pastoralists and seek to involve actors from governmental agencies for humans, animals, and the environment, for the improved health of children in pastoralist communities. |
| Generalisability | | | 21 | | Discuss the generalisability (external validity) of the study results | | | | 22 | - “In addition, this study adds to the limited body of research on the health of pastoralist communities living in the ESRS and in the horn of Africa, the latter geographic zone encompassing an estimated 20-30 million humans living a pastoral lifestyle [21,22].” |
| Other information | | | | | | | | | | |
| Funding | | | 22 | | Give the source of funding and the role of the funders for the present study and, if applicable, for the original study on which the present article is based | | | | 1 | - “This study received financial support from the Swiss Agency for Development and Cooperation (SDC) (AM, no. 7F-09057.01.02), the Nutricia Research Foundation (PV, no. 2019-20), the Forschungsfonds of the University of Basel, the SNSF Return Grant (PV, no. [P3P3PA_177877](https://www.mysnf.ch/grants/grant.aspx?id=48c50816-aa5b-41a1-9d57-25b37f007e26)) and the Eccellenza Fellowship (PV, no. [PCEFP3_194545](https://www.mysnf.ch/grants/grant.aspx?id=f5dedcbe-4c2d-4f95-bb00-c05870b123b7)). The funders had no role in study design, data collection and analysis, decision to publish, or preparation of the manuscript. |

*Give information separately for cases and controls in case-control studies and, if applicable, for exposed and unexposed groups in cohort and cross-sectional studies.

**Note:** An Explanation and Elaboration article discusses each checklist item and gives methodological background and published examples of transparent reporting. The STROBE checklist is best used in conjunction with this article (freely available on the Web sites of PLoS Medicine at http://www.plosmedicine.org/, Annals of Internal Medicine at http://www.annals.org/, and Epidemiology at http://www.epidem.com/). Information on the STROBE Initiative is available at [www.strobe-statement.org](http://www.strobe-statement.org).

# References

1. WHO Multicentre Growth Reference Study Group. Assessment of differences in linear growth among populations in the WHO Multicentre Growth Reference Study. Acta Paediatr Suppl. 2006 Apr;450:56–65.

2. Hartung C, Lerer A, Anokwa Y, Tseng C, Brunette W, Borriello G. Open Data Kit: Tools to Build Information Services for Developing Regions. In: Proceedings of the 4th ACM/IEEE International Conference on Information and Communication Technologies and Development [Internet]. New York, NY, USA: Association for Computing Machinery; 2010. (ICTD ’10). Available from: https://doi.org/10.1145/2369220.2369236

3. Katz N, Chaves A, Pellegrino J. A simple device for quantitative stool thick-smear technique in Schistosomiasis mansoni. Rev Inst Med Trop Sao Paulo. 1972 Dec;14(6):397–400.

4. Osman KA, Zinsstag J, Tschopp R, Schelling E, Hattendorf J, Umer A, et al. Nutritional status and intestinal parasites among young children from pastoralist communities of the Ethiopian Somali region. Matern Child Nutr. 2020 Jul;16(3):e12955.

5. R Core Team. R: A language and environment for statistical computing. [Internet]. Vienna, Austria: R Foundation for Statistical Computing; 2013. Available from: http://www.R-project.org/

6. Rich B. table1: Tables of Descriptive Statistics in HTML. [Internet]. 2021. Available from: https://CRAN.R-project.org/package=table1

7. Højsgaard S, Halekoh U, Yan J. The R Package geepack for Generalized Estimating Equations. Journal of Statistical Software. 2006;15:1–11.

8. Bursac Z, Gauss CH, Williams DK, Hosmer DW. Purposeful selection of variables in logistic regression. Source Code for Biology and Medicine. 2008 Dec 16;3(1):17.

9. Stensvold CR, Nielsen HV. Comparison of Microscopy and PCR for Detection of Intestinal Parasites in Danish Patients Supports an Incentive for Molecular Screening Platforms. Journal of Clinical Microbiology. 2012 Feb;50(2):540–1.

10. Utzinger J, Botero-Kleiven S, Castelli F, Chiodini PL, Edwards H, Köhler N, et al. Microscopic diagnosis of sodium acetate-acetic acid-formalin-fixed stool samples for helminths and intestinal protozoa: a comparison among European reference laboratories. Clinical Microbiology and Infection. 2010 Mar 1;16(3):267–73.

11. Zinsstag J, Schelling E, Crump L, Whittaker M, Tanner M, Stephen C, editors. One Health: The Theory and Practice of Integrated Health Approaches [Internet]. 2nd ed. 2020 [cited 2021 Aug 31]. 464 p. Available from: https://www.cabi.org/bookshop/book/9781789242577/

12. CDC. Zoonotic Diseases | One Health | CDC [Internet]. 2021 [cited 2021 Aug 31]. Available from: https://www.cdc.gov/onehealth/basics/zoonotic-diseases.html

13. Gizaw Z, Yalew AW, Bitew BD, Lee J, Bisesi M. Fecal indicator bacteria along multiple environmental exposure pathways (water, food, and soil) and intestinal parasites among children in the rural northwest Ethiopia. BMC Gastroenterol. 2022 Feb 27;22(1):84.

14. Barnes AN, Davaasuren A, Baasandavga U, Lantos PM, Gonchigoo B, Gray GC. Zoonotic enteric parasites in Mongolian people, animals, and the environment: Using One Health to address shared pathogens. PLoS Negl Trop Dis. 2021 Jul 8;15(7):e0009543.

15. Lee SC, Ngui R, Tan TK, Roslan MA, Ithoi I, Mahdy MAK, et al. Understanding Giardia infections among rural communities using the one health approach. Acta Tropica. 2017 Dec 1;176:349–54.

16. Pawestri AR, Thima K, Leetachewa S, Maneekan P, Deesitthivech O, Pinna C, et al. Seasonal prevalence, risk factors, and One Health intervention for prevention of intestinal parasitic infection in underprivileged communities on the Thai-Myanmar border. International Journal of Infectious Diseases. 2021 Apr 1;105:152–60.

17. Angora EK, Vangraefschepe A, Allienne JF, Menan H, Coulibaly JT, Meïté A, et al. Population genetic structure of Schistosoma haematobium and Schistosoma haematobium × Schistosoma bovis hybrids among school-aged children in Côte d’Ivoire. Parasite. 2022;29:23.

18. Hughes SD, Woods WJ, O’Keefe KJ, Delgado V, Pipkin S, Scheer S, et al. Integrating Phylogenetic Biomarker Data and Qualitative Approaches: An Example of HIV Transmission Clusters as a Sampling Frame for Semistructured Interviews and Implications for the COVID-19 Era. Journal of Mixed Methods Research. 2021 Jul 1;15(3):327–47.

19. Kaur M. Application of Mixed Method Approach in Public Health Research. Indian J Community Med. 2016;41(2):93–7.

20. Zinsstag J, Schelling E, Waltner-Toews D, Tanner M. From “one medicine” to “one health” and systemic approaches to health and well-being. Prev Vet Med. 2011 Sep 1;101(3–4):148–56.

21. Horn of Africa - Multi-Sectoral Interventions in Pastoralist Communities Fact Sheet #2, Fiscal Year (FY) 2005 - Djibouti | ReliefWeb [Internet]. [cited 2022 Jun 16]. Available from: https://reliefweb.int/report/djibouti/horn-africa-multi-sectoral-interventions-pastoralist-communities-fact-sheet-2-fiscal

22. Mkutu K. Pastoralism and Conflict in the Horn of Africa. Saferworld; 2001.
