# Supplementary material for: Prevalence and associated risk factors of intestinal parasitic infections among children in pastoralist and agro-pastoralist communities in the Adadle woreda of the Somali Regional State of Ethiopia
Source: PLoS Negl Trop Dis. 2023 Jul 3;17(7):e0011448. doi: 10.1371/journal.pntd.0011448 (PMC10348586; doi:10.1371/journal.pntd.0011448)
Supplement: S3 File — R Markdown master file for reproducing results. (DOCX) [file pntd.0011448.s007.docx]

**R Master File:** Markdown code for the replication of data analysis in the manuscript “Prevalence and associated risk factors of intestinal parasitic infections among children in pastoralist and agro-pastoralist communities in the Adadle woreda of the Somali Region of Ethiopia.” Lanker and Muhummed et al, 2023.

**Instructions for use:**

1. Open R Statistical software version 4.0.4 [1]
2. Set your working directory to include access to **S2_file_data_manuscript.csv**
3. Open a new R Markdown file, choose Default Output Format: Word
4. Delete pre-written content and replace with the entirety of the below code (highlighted in gray)
5. Make sure to download all external packages, including the gtsummary package [2] and the geepack package [3]
6. Knit to Word

**R Markdown Code:**

---

title: "Lanker Muhummed et al R Tables & Figures Master"

author: "Created by: Kayla C. Lanker"

date: "2023-Mar-31"

output: word_document

---

```{r setup, include=FALSE}

library(tidyverse)

library(readxl)

library(plyr)

library(dplyr)

library(geepack)

library(gtsummary)

library(gt)

library(flextable)

library(table1)

library(Hmisc)

knitr::opts_chunk$set(echo = FALSE,warning=FALSE, message=FALSE)

```

```{r, echo = FALSE}

####Load data

data_manuscript <- read_csv("S2_file_data_manuscript.csv")

data_analysis <- subset(data_manuscript, !is.na(parasite_num_bi)) #for analyses with N=366, only parasitology prevalence

```

```{r, echo = FALSE}

####Labels

Hmisc::label(data_analysis$group) <- "Group"

Hmisc::label(data_analysis$parasite_num_bi) <- "Parasite detected"

Hmisc::label(data_analysis$parasite_num) <- "Parasite number"

Hmisc::label(data_analysis$parasite_type) <- "Parasite type"

Hmisc::label(data_analysis$kk_para_type) <- "Helminth type"

Hmisc::label(data_analysis$wet_mount) <- "Protozoa type"

Hmisc::label(data_analysis$kk_bi) <- "Helminths"

Hmisc::label(data_analysis$wet_mount_bi) <- "Protozoa"

Hmisc::label(data_analysis$giardia_bi) <- " Giardia intestinalis"

Hmisc::label(data_analysis$histolytica_bi) <- " Entamoeba histolytica/dispar"

Hmisc::label(data_analysis$ascaris_bi) <- " Ascaris lumbricoides"

Hmisc::label(data_analysis$hookworm_bi) <- " Hookworm"

Hmisc::label(data_analysis$hnana_bi) <- " Hymenolepis nana"

Hmisc::label(data_analysis$stool_consistency) <- "Stool consistency"

Hmisc::label(data_analysis$gender) <- "Sex"

Hmisc::label(data_analysis$age_months) <- "Age"

Hmisc::label(data_analysis$age_yr) <- "Age"

Hmisc::label(data_analysis$weight) <- "Weight"

Hmisc::label(data_analysis$height) <- "Height"

Hmisc::label(data_analysis$wasting_st) <- "Wasting status"

Hmisc::label(data_analysis$stunting) <- "Stunting status"

Hmisc::label(data_analysis$muac) <- "MUAC"

Hmisc::label(data_analysis$muac_low) <- "Low MUAC (<12.5 cm)"

Hmisc::label(data_analysis$vax_done) <- "Vaccination status"

Hmisc::label(data_analysis$first_lang_hh) <- "Household language"

Hmisc::label(data_analysis$hhh_religion)<- "Household religion"

Hmisc::label(data_analysis$hhh_grade_gr) <- "Head of household grades completed"

Hmisc::label(data_analysis$hhh_literate) <- "Head of household literacy"

Hmisc::label(data_analysis$hhh_lit_bi) <- "Head of household is literate"

Hmisc::label(data_analysis$chmo_grade_gr) <- "Mother of child grades completed"

Hmisc::label(data_analysis$chmo_literate) <- "Mother of child literacy"

Hmisc::label(data_analysis$chmo_lit_bi) <- "Mother of child is literate"

Hmisc::label(data_analysis$hhassets_s67) <- "Household has a mobile phone"

Hmisc::label(data_analysis$drinkwater_source_cat) <- "Source of drinking water"

Hmisc::label(data_analysis$toilet_cat) <- "Toilet type"

Hmisc::label(data_analysis$toilet_share) <- "Shared Toilet"

Hmisc::label(data_analysis$waste_dispose_bi) <- "Waste disposal"

Hmisc::label(data_analysis$dwater_treat) <- "Treatment of Water"

Hmisc::label(data_analysis$child_wash_hands_cat) <- "Child hand washing method"

Hmisc::label(data_analysis$hh_soap_bi) <- "Household has Soap"

Hmisc::label(data_analysis$hhanimal_cum) <- "Household herd size"

Hmisc::label(data_analysis$house_animal) <- "Animals kept inside house"

Hmisc::label(data_analysis$hhanimal_cattle) <- "Owns cattle"

Hmisc::label(data_analysis$hhanimal_cattle_c) <- "Number of cattle"

Hmisc::label(data_analysis$hhanimal_camel) <- "Owns camels"

Hmisc::label(data_analysis$hhanimal_camel_c) <- "Number of camels"

Hmisc::label(data_analysis$hhanimal_goat) <- "Owns goats"

Hmisc::label(data_analysis$hhanimal_goat_c) <- "Number of goats"

Hmisc::label(data_analysis$hhanimal_sheep) <- "Owns sheep"

Hmisc::label(data_analysis$hhanimal_sheep_c) <- "Number of sheep"

Hmisc::label(data_analysis$hhanimal_donkey) <- "Owns donkeys"

Hmisc::label(data_analysis$hhanimal_donkey_c) <- "Number of donkeys"

Hmisc::label(data_analysis$hhanimal_chicken) <- "Owns chickens"

Hmisc::label(data_analysis$hhanimal_chicken_c) <- "Number of chickens"

Hmisc::label(data_analysis$breastfeed) <- "Was child breastfed?"

Hmisc::label(data_analysis$breastfeed_time) <- "Breastfeed time"

Hmisc::label(data_analysis$breastfeed_time_c) <- "Breastfeed time"

Hmisc::label(data_analysis$breastfeed_ex6mo) <- "Exclusively breastfed for 6 months"

Hmisc::label(data_analysis$other_milk) <- "Complementary milk"

Hmisc::label(data_analysis$other_milk_when) <- "Complementary milk starting when"

Hmisc::label(data_analysis$other_milk_when_c) <- "Complementary milk starting when"

Hmisc::label(data_analysis$comp_food_when) <- "Complementary food starting when"

Hmisc::label(data_analysis$comp_food_when_c) <- "Complementary food starting when"

Hmisc::label(data_analysis$first_birth_when) <- "Age of mother at first birth"

Hmisc::label(data_analysis$num_children) <- "Number of children of mother"

data_analysis$first_lang_hh <- mapvalues(data_analysis$first_lang_hh,1,"Somali")

data_analysis$hhh_religion <- mapvalues(data_analysis$hhh_religion,1,"Islam")

##Variable Units

units(data_analysis$age_months) <- "months"

units(data_analysis$weight) <- "kg"

units(data_analysis$height) <- "cm"

units(data_analysis$muac) <- "cm"

data_analysis2 <- subset(data_analysis, !is.na(age_yr)) #for analyses with N=345, perfect match ODK + parasitology

```

```{r, echo = FALSE}

# function for estimating the confidence interval for the odds ratio

OR <- function(gee){

for(i in 2:nrow(coef(summary(gee))))

print(round(exp(coef(summary(gee))[i,1] + coef(summary(gee))[i,2]*c(0,-1.96,1.96)),2))

}

#kebele_code - cluster id - change it from character to factor

data_analysis2$kebele_code <- as.factor(data_analysis2$kebele_code)

#ordering the data

data_analysis2 <- data_analysis2[order(data_analysis2$kebele_code),]

my_geeglm <- function(formula, data, id, ...) {

# capture id input (since it's unquoted)

id <- rlang::enexpr(id)

# keep complete cases amoung the variables needed in the model

data <-

select(data, all_of(all.vars(formula)), !!id) %>%

dplyr::filter(complete.cases(.))

# build GEE model

rlang::inject(

geepack::geeglm(

formula = formula,

data = data,

id = !!id, # inserting unquoted id column name

...))}

```

\newpage

```{r, echo=FALSE}

data_analysis1 <- subset(data_analysis, !is.na(hhh_religion)) #for analyses with N=358, household analyses

```

## **Manuscript Tables 1-6 & Figure 2** {#css_id}

### **Table 1.** Characteristics of (agro-) pastoralist children aged 2 - 5 years living in Adadle Woreda, Somali region, Ethiopia. {.css_class}

```{r, echo=FALSE}

#Table 1 N=345 use data_analysis2

t1 <-data_analysis2 %>%

select(age_yr, gender, vax_done, breastfeed_ex6mo, other_milk_when_c, comp_food_when_c) %>%

tbl_summary(missing = "ifany", digits = list(everything() ~ c(0, 1))) %>% bold_labels()

t1 %>% as_flex_table() %>%

bold(part="header")

```

\newpage

### **Table 2.** Anthropometric characteristics of agro-pastoralist and pastoralist children aged 2 - 5 years living in Adadle woreda, Somali region, Ethiopia. {.css_class}

```{r, echo=FALSE}

#Table 2 N=345 use data_analysis2

t2 <- data_analysis2 %>%

select(height,weight,hfaz,stunting, wfhz,wasting_st,wfaz,underweight,muac,muac_low,group) %>%

tbl_summary(by= group, missing = "ifany") %>%

add_overall(last=TRUE) %>% bold_labels()

t2 %>% as_flex_table(

include = everything()) %>%

bold(part="header")

```

\newpage

### **Table 3.** Household WASH characteristics of agro-pastoralist and pastoralist children aged 2 - 5 years living in Adadle woreda, Somali region, Ethiopia. {.css_class}

```{r}

#Table 3 N=358 use data_analysis1

t3 <-data_analysis1 %>%

select(drinkwater_source_cat, dwater_treat, toilet_cat, toilet_share, waste_dispose_bi, hh_soap_bi,child_wash_hands_cat,group) %>%

tbl_summary(by=group, missing = "ifany", digits = list(everything() ~ c(0, 1))) %>% add_overall(last=TRUE) %>% bold_labels()

t3 %>% as_flex_table() %>%

bold(part="header")

```

\newpage

### **Table 4.** Prevalence of intestinal parasitic infections during the 2021 wet season in agro-pastoralist and pastoralist children aged 2 - 5 years living in Adadle woreda, Somali region, Ethiopia. {.css_class}

```{r}

#Table 4 N=366 use data_analysis

data_analysis$parasite_num <- mapvalues(data_analysis$parasite_num, from=c(0,1,2), to=c("Parasite-free","Single-parasite","Poly-parasite"))

data_analysis$parasite_num <- factor(data_analysis$parasite_num, level=c("Parasite-free","Single-parasite","Poly-parasite"))

label(data_analysis$parasite_num) <- "Overall"

#Overall

tbl_a <-

data_analysis %>%

select(group, parasite_num) %>%

tbl_summary(by = group, missing = "no", digits = list(everything() ~ c(0, 1))) %>%

add_overall(last=TRUE) %>% bold_labels()

# Protozoa

tbl_b <-

data_analysis %>%

select(group, giardia_bi, histolytica_bi) %>%

tbl_summary(by = group, missing = "ifany", digits = list(everything() ~ c(0, 1)))%>%

add_overall(last=TRUE) %>% italicize_labels()

# Helminths

tbl_c <-

data_analysis %>%

select(group, ascaris_bi,hookworm_bi,hnana_bi) %>%

tbl_summary(by = group, missing="ifany", digits = list(everything() ~ c(0, 1))) %>%

add_overall(last=TRUE)%>% italicize_labels()

#stack tables

t4 <- tbl_stack(list(tbl_a, tbl_b, tbl_c), group_header = c("", "Protozoa", "Helminths")) %>%

as_gt() %>%

gt::tab_style(

style = gt::cell_text(weight = "bold"),

locations = gt::cells_row_groups(groups = everything()))

t4

```

\newpage

### **Table 5.** Multivariate Analysis of *Giardia lamblia* in agro-pastoralist and pastoralist children aged 2 - 5 years living in Adadle woreda, Somali region, Ethiopia. {.css_class}

```{r, echo=FALSE, message=FALSE}

#Table 5 N=345 use data_analysis2

#univariate

t5_1 <- data_analysis2 %>%

select(kebele_code, group, gender, age_yr, drinkwater_source_cat, toilet_share, hhanimal_cattle_c, hhanimal_chicken, giardia_bi) %>%

tbl_uvregression(

method = my_geeglm,

y = giardia_bi,

include = -`kebele_code`,

method.args = list(family = binomial,id = `kebele_code`,corstr = "independence",scale.fix = TRUE)

,exponentiate = TRUE, hide_n = TRUE, pvalue_fun = ~style_pvalue(.x, digits = 2)) %>%

#add_significance_stars(pattern = "{estimate} ({conf.low}, {conf.high}){stars}", hide_se = TRUE) %>%

modify_header(estimate ~ "**OR**") %>%

bold_labels() %>%

add_nevent(location = "level") %>%

add_n(location = "level") %>%

# adding event rate

modify_table_body(

~ .x %>%

dplyr::mutate(

stat_nevent_rate =

ifelse(

!is.na(stat_nevent),

paste0(style_sigfig(stat_nevent / stat_n, scale = 100)),

NA), .after = stat_nevent)) %>%

# merge the colums into a single column

modify_column_merge(

pattern = "{stat_nevent} / {stat_n} ({stat_nevent_rate})",

rows = !is.na(stat_nevent)

) %>%

# update header to event rate

modify_header(stat_nevent = "**n/N (% Positive)**")

#multivariate

mf1 <- formula(giardia_bi ~ group + gender + age_yr + drinkwater_source_cat + toilet_share + hhanimal_cattle_c + hhanimal_chicken)

g1 <- my_geeglm(formula = mf1, family = binomial, data=data_analysis2,

id=kebele_code, corstr = "independence", std.err="san.se")

t5_2 <- tbl_regression(g1,exponentiate = TRUE, pvalue_fun = ~style_pvalue(.x, digits = 2)) %>%

#add_significance_stars(pattern = "{estimate} ({conf.low}, {conf.high}){stars}",hide_se = TRUE) %>%

modify_header(estimate ~ "**aOR**") %>%

bold_labels()

tbl_merge1 <- tbl_merge(tbls = list(t5_1, t5_2), tab_spanner = c("**Univariate**", "**Multivariate**"))

tbl_merge1 %>% as_flex_table() %>%

bold(part="header")

```

\newpage

### **Table 6.** Multivariate Analysis of *Ascaris lumbricoides* in agro-pastoralist and pastoralist children aged 2 - 5 years living in Adadle woreda, Somali region, Ethiopia. {.css_class}

```{r, echo=FALSE, message=FALSE}

#Table 6 N=345 use data_analysis2

#bivariate

t6_1 <- data_analysis2 %>%

select(kebele_code, group, gender, age_yr, drinkwater_source_cat,toilet_share, hhanimal_cattle_c, hhanimal_chicken, ascaris_bi) %>%

tbl_uvregression(

method = my_geeglm,

y = ascaris_bi,

include = -`kebele_code`,

method.args = list(family = binomial,id = `kebele_code`,corstr = "independence",scale.fix = TRUE)

,exponentiate = TRUE, hide_n = TRUE, pvalue_fun = ~style_pvalue(.x, digits = 2)) %>%

#add_significance_stars(pattern = "{estimate} ({conf.low}, {conf.high}){stars}",hide_se = TRUE) %>%

modify_header(estimate ~ "**OR (95% CI)**") %>%

bold_labels() %>%

add_nevent(location = "level") %>%

add_n(location = "level") %>%

# adding event rate

modify_table_body(

~ .x %>%

dplyr::mutate(

stat_nevent_rate =

ifelse(

!is.na(stat_nevent),

paste0(style_sigfig(stat_nevent / stat_n, scale = 100)),

NA), .after = stat_nevent)) %>%

# merge the colums into a single column

modify_column_merge(

pattern = "{stat_nevent} / {stat_n} ({stat_nevent_rate})",

rows = !is.na(stat_nevent)

) %>%

# update header to event rate

modify_header(stat_nevent = "**n/N (% Positive)**")

#multivariate

mf2 <- formula(ascaris_bi ~ group + gender + age_yr + drinkwater_source_cat + toilet_share + hhanimal_cattle_c + hhanimal_chicken)

g2 <- my_geeglm(formula = mf2, family = binomial, data=data_analysis2,

id=kebele_code, corstr = "independence", std.err="san.se")

t6_2 <- tbl_regression(g2,exponentiate = TRUE, pvalue_fun = ~style_pvalue(.x, digits = 2)) %>%

#add_significance_stars(pattern = "{estimate} ({conf.low}, {conf.high}){stars}",hide_se = TRUE) %>%

modify_header(estimate ~ "**aOR (95% CI)**") %>%

bold_labels()

tbl_merge2 <- tbl_merge(tbls = list(t6_1, t6_2), tab_spanner = c("**Univariate**", "**Multivariate**"))

tbl_merge2 %>% as_flex_table() %>%

bold(part="header")

```

\newpage

### **Figure 2: Average household animal herd makeup of agro-pastoralist and pastoralist children aged 2 - 5 years of age living in Adadle woreda, Somali region, Ethiopia.**

####NOTE: This is data from which Figure 2 was created in Excel v16.67. Use the chart titled "Sunburst".

```{r, echo = FALSE, require(dplyr)}

#Figure2 N=358 use data_analysis1

animals_mn <- data_analysis1 %>% dplyr::group_by(group) %>%

dplyr::summarise(mean_cattle=mean(hhanimal_cattle_n),

mean_camel= mean(hhanimal_camel_n),

mean_goat= mean(hhanimal_goat_n),

mean_sheep= mean(hhanimal_sheep_n),

mean_donkey= mean(hhanimal_donkey_n),

mean_chicken= mean(hhanimal_chicken_n),

.groups = 'drop') %>% as.data.frame()

animals_sd <- data_analysis1 %>% dplyr::group_by(group) %>%

dplyr::summarise(sd_cattle=sd(hhanimal_cattle_n),

sd_camel= sd(hhanimal_camel_n),

sd_goat= sd(hhanimal_goat_n),

sd_sheep= sd(hhanimal_sheep_n),

sd_donkey= sd(hhanimal_donkey_n),

sd_chicken= sd(hhanimal_chicken_n),

.groups = 'drop') %>% as.data.frame()

animals_n <- data_analysis1 %>% dplyr::count(group)

animals_n <- animals_n %>% mutate(sqrt_n = sqrt(n))

animals_sd$group <- as.factor(animals_sd$group)

animals_sd_n <- merge(animals_sd, animals_n, by=c("group"),all.x = TRUE, all.y = TRUE)

animals_95ci <- animals_sd_n %>%

mutate(ci_cattle = (sd_cattle/sqrt_n)*1.96) %>%

mutate(ci_camel = (sd_camel/sqrt_n)*1.96) %>%

mutate(ci_goat = (sd_goat/sqrt_n)*1.96) %>%

mutate(ci_sheep = (sd_sheep/sqrt_n)*1.96) %>%

mutate(ci_donkey = (sd_donkey/sqrt_n)*1.96) %>%

mutate(ci_chicken = (sd_chicken/sqrt_n)*1.96)

animals_95ci <- subset(animals_95ci, select=-c(sd_cattle,sd_camel,sd_goat,sd_sheep,sd_donkey,sd_chicken, n, sqrt_n))

animals_mn

animals_95ci

```

\newpage

## **Supporting tables** {#css_id}

### **S1 Table.** Sociodemographic household characteristics of agro-pastoralist and pastoralist children aged 2 - 5 years living in Adadle woreda, Somali region, Ethiopia. {.css_class}

```{r}

#S1Table N=358 use data_analysis1

ts1 <-data_analysis1 %>%

select(first_lang_hh, hhh_religion, hhh_lit_bi, chmo_lit_bi, hhassets_s67, group) %>%

tbl_summary(by= group, missing = "no", digits = list(everything() ~ c(0, 1))) %>% add_overall(last=TRUE) %>% bold_labels()

ts1 %>% as_flex_table() %>%

bold(part="header")

```

\newpage

### **S2 Table.** Household animal herds of agro-pastoralist and pastoralist children aged 2 - 5 years of age living in Adadle woreda, Somali region, Ethiopia. {.css_class}

```{r}

#S2Table N=358 use data_analysis1

ts2 <-data_analysis1 %>%

select(hhanimal_cum, house_animal, hhanimal_cattle_c, hhanimal_camel_c, hhanimal_goat_c, hhanimal_sheep_c,hhanimal_donkey_c,hhanimal_chicken_c,group) %>%

tbl_summary(by=group, missing = "no", digits = list(everything() ~ c(0, 1))) %>% add_overall(last=TRUE) %>% bold_labels()

ts2 %>% as_flex_table() %>%

bold(part="header")

```

# References

1. R Core Team. R: A language and environment for statistical computing. [Internet]. Vienna, Austria: R Foundation for Statistical Computing; 2013. Available from: http://www.R-project.org/

2. Sjoberg DD, Whiting K, Curry M, Lavery JA, Larmarange J. Reproducible Summary Tables with the gtsummary Package. The R Journal. 2021;13(1):570–80.

3. Højsgaard S, Halekoh U, Yan J. The R Package geepack for Generalized Estimating Equations. Journal of Statistical Software. 2006;15:1–11.
