# Supplementary material for: Prevalence and associated risk factors of intestinal parasitic infections among children in pastoralist and agro-pastoralist communities in the Adadle woreda of the Somali Regional State of Ethiopia
Source: PLoS Negl Trop Dis. 2023 Jul 3;17(7):e0011448. doi: 10.1371/journal.pntd.0011448 (PMC10348586; doi:10.1371/journal.pntd.0011448)
Supplement: Attachment — Submitted filename: ResponseLetterReviewersPlosNTD_010423.pdf [file pntd.0011448.s010.pdf]

UNIL | Université de Lausanne
Département de microbiologie fondamentale
bâtiment Biophore bureau BIO-2310.1::02
CH-1015 Lausanne

Prof. Pascale Vonaesch
Department of Fundamental Microbiology
University of Lausanne
Campus UNIL-Sorge
1015 Lausanne

Lausanne, the 1st of April 2023

Subject: Point-to-point reply for the revisions regarding the manuscript in Plos NTD

Dear editor, dear reviewers

We would like to thank the reviewers for their thorough review, comments and criticisms for the manuscript entitled "**Prevalence and associated risk factors of intestinal parasitic infections among children in pastoralist and agro-pastoralist communities in the Adadle woreda of the Somali Region of Ethiopia**", which allowed to significantly improve the quality of the manuscript.

In the point-to-point reply provided below, we included our answers to the points raised by both reviewers and describe the overall changes made on the revised manuscript. We also submit the new version of the manuscript including track changes, a clean version as well as the revised Supplement.

We hope the manuscript in its revised form is now acceptable for publication in Plos NTD.

Yours sincerely,

Pascale Vonaesch and co-authors

Faculté de biologie et de médecine
Département de microbiologie fondamentale

Tél.+41 21 692 56 09 | Fax.+41 21 692 56 05 | pascale.vonaesch@unil.ch

Point-to-point reply

Methods

Reviewer #1:

1. The targeted population is small and restricted to Adadle woreda of the Somali Region. Woreda is the district level and kebele is the smallest administrative unit of Ethiopia. Adadle woreda is one of 10 districts of Shabelle Zone of the Somali Region State of Ethiopia.
   Thank you for your feedback. We now clarified this point to the reader and indicate this clearly in several sections of the revised manuscript.

2. Prevalence and risk factors of wasting, stunting and underweight were presented; however, these were not indicated among the objectives of the study.
   Thank you for this justified critique. Since our sampling included selection of severely wasted and stunted children, we have decided to remove all nutritional status prevalence estimates as well as univariate and multivariate supplementary tables and text in the results and discussion related to nutritional status measures (wasting, stunting, and underweight).

3. The methods used are appropriate and clearly described.
   Thank you for your feedback.

Reviewer #2: (No Response)

Reviewer #3: The objective of the study is well articulated and match the study design. The population studied is clearly described and is well suited for the study. The sample size is robust and appropriate for the for the statistics carried out.
Thank you for your feedback.

Results

Reviewer #1:

1. Table 6: What is the rationale of "Owns cattle" variable grouping and cut-offs? It seems that difference between NO (10%) and YES (14.9%) might be not significant.
   Thank you for this critique. The grouping and cut offs for the categorical variable "owns cattle" were based on the median of the original numerical variable. We have added information on categorization of numerical variables in the statistical analysis section, lines 222-223 in the clean version of the manuscript, "Categorization of numerical variables, such as ownership of cattle, were based on the median.". The reviewer is right that the effect would not become significant, if we would consider the binary variable cattle ownership yes/no instead of the 3 categories (OR 1.5, CI 0.8 to 3.1, p = 0.2).

2. Line 151: "All screened children who were severely wasted based on their weight for height z-score (WHZ < -2) or severely stunted based on their height for age z-score (HAZ< -2) were automatically included in the study." How many severely wasted or severely stunted children included?
   Thank you for this question and we agree it should be mentioned in the manuscript. Accordingly, we have added the following section in the results, lines 235-237 in the clean version of the manuscript: "During the screening process, 7 children presented as severely stunted, 27 as severely wasted, and 3 as severely stunted and severely wasted, all of whom were included in the study."

3. Lines 248-250: How many severely wasted or severely stunted children included? Because these children were purposively selected, analysis should be adjusted.
   The reviewer raised an important point. It is correct that severely wasted and stunted children had a higher selection probability compared to other children and an analysis including sampling weights could account for this. Unfortunately, we don't have the required information to assign correct sampling weights for the "non severe" children. However, we take the reviewer's concern seriously. The strongest bias will occur in the prevalence estimates. Therefore, we removed all nutritional status prevalence estimates as well as univariate and multivariate supplementary tables and text in the results and discussion related to nutritional status measures (wasting, stunting, and underweight).

4. Table 1: please revise the numbers; e.g. 335 for Complementary food starting; 341 for Complementary milk starting; 338 for vaccination status, etc. It is stated that 345 children had a complete questionnaire and parasitology report (line 223).
   Thank you for your attention to detail and for pointing this out. We have added the "Unknowns" to Table 1, so it is clear to the reader. These data points were inputted as "99, unknown", therefore they do not constitute as missing values. The wording has also been changed in the revised manuscript to "completed a questionnaire" instead of "complete questionnaire".

5. Table 5: Why some factors are not included in analysis of Ascaria? E.g. Drinking water source and ownership of chicken. All factors should be included regardless significant association. Similarly, some variables in Table 6 are not included in analysis of Giardia; e.g. shared toilet and household owns soap.
   Thank you for this question and suggestion. We indeed hadn't adequately described the variable selection process in the original manuscript, which was a combination of known confounder from the literature, expert opinion and variables showing associations in the bivariable analyses. We follow the suggestion of the reviewer and have included the same variables for both *Ascaria lumbricoides* and *Giardia intestinalis* in the analysis.

6. Tables 5 and 6: add columns for P values for bivariate and multivariate. Although it can be judged from the 95%CI, but it still useful.
   We added P values as suggested but we would like to point out that PLOS NTD requests that observational studies should be presented according to the STROBE guidelines. While the guideline does not specifically prohibit p values, it is discouraged.

Reviewer #2: (No Response)

Reviewer #3: The results are well presented with appropriate figures and tables.
Thank you for your feedback.

Conclusions

Reviewer #1:

1. Lines 44-45: "employing a One Health approach to treat children and animals for parasitic infections are important" this is not supported by the findings. Despite of significant associations detected, the study design does not allow causal inference. This is also applied to discussion section. This is also applied for main conclusion section.
   Thank you for this suggestion and we agree that this is formulated incorrectly. We have rewritten this sentence in the abstract, lines 46-47 in the clean version of the manuscript, which now reads as follows: "Improving access to safe water, sanitation, and hygiene services in Adadle and employing a One Health

approach in future studies on parasitic infections, would likely improve the health of children living in (agro-)pastoralist communities in Adadle and ESRS". In the discussion the following change was made to convey the sentence as a suggestion instead of as a finding: Discussion, lines 456-458 in the clean, revised manuscript, "Given that pastoralists live in close connection with their animals and their shared environment, we recommend future studies in pastoralist communities on IPIs consider and implement a One Health approach [9]."

2. Discussion about the association found between owning chicken and Giardia infection lacks in depth explanation.
   Thank you for your critique. The following addition has been made in the discussion, lines 453-456 in the clean, revised manuscript: " In addition, free-range chickens may also be apt disseminators of *G. intestinalis* cysts throughout the communal environment, other households, and other animals, as there are not often bound or fenced in [60,61]."

3. Lines 454-467: this paragraph about nutritional status can be removed. It is recommended to add related variables to analysis on stunting and wasting and then discuss the association between IPIs and nutritional status. Table 1 S shows negative association between Giardia and Ascaris with wasting; wasting can be added as a variable in Tables 5 and 6. Malnutrition and IPIs are coexisted problems and one is causative for the other.
   Thank you for this point. We removed the nutritional status prevalence estimates as well as univariate and multivariate supplementary tables and text in the results and discussion related to nutritional status measures (see also our response to reviewer #1 point 3). With respect to the other point raised – to include wasting and stunting in the regression models, we had several discussions in our team. Our statistician recommended not to include these variables in the model because – as the reviewer pointed out correctly – malnutrition and intestinal parasites are closely linked, and the direction of the effect is not entirely clear. We assume that it is more likely that the parasites causes/contributes malnutrition than the other way round. In this case an inclusion of the variable in the model would lead to collider bias. For the reviewer's information, we tested wasting and stunting in our model, but both were non-significant in the multivariate analysis.

Reviewer #2: (No Response)

Reviewer #3: The conclusion in this study is supported by the data presented and limitations of the study stated. The public health importance is clearly emphasized. Thank you for your feedback.

Editorial and Data Presentation Modifications?

Reviewer #1:

1. The title should be rephrased. The subjects and targeted populations are unclear. It should be "…… among young children in pastoralist and agro-pastoralist populations (or can be communities) in the Somali Region of Ethiopia". This is also applied for related sentences in the text.
   Thank you for this critique and we agree. We have changed the title to the following: "Prevalence and associated risk factors of intestinal parasitic infections among children in pastoralist and agro-pastoralist communities in the Adadle woreda of the Somali Region of Ethiopia".

2. Giardia lamblia: lamblia is an old name; you may use Giardia intestinalis throughout the manuscript.
   We agree with this and have incorporated your suggestion throughout the manuscript.

3. Line 34: state the overall prevalence first; i.e. those infected with at least one parasite species.

Thank you for pointing this out. We agree with this comment. Therefore, we have added the following in the abstract, line 36: "Overall IPI prevalence was 35%".

4. Lines 37-43: sentences about risk factors can be rephrased. Just indicate the risk factors with respective statistics (aOR & 95%CI); no need to mention the counterpart groups.
   Thank you for this suggestion. We have changed these sentences in the Abstract, lines 40-45: " *G.* intestinalis infection was associated with drinking water sourced from the river (aOR 15.6, 95%CI 6.84, 35.4) and from collected rainwater (aOR 9.48, 95%CI 3.39, 26.5), with toilet sharing (aOR 2.93, 95%CI 1.36, 6.31) and with household ownership of cattle (1-5 cattle: aOR 1.65, 95%CI 1.13, 2.41; 6+ cattle: aOR 2.07, 95%CI 1.33, 3.21) and chickens (aOR 3.80, 95%CI 1.77, 8.17). *A. lumbricoides* infection was associated with children 36-47 months old (aOR 1.92, 95%CI 1.03, 3.58). "

5. Line 78: "Most human intestinal parasites require an intermediate animal host to complete their lifecycle," this is not accurate; at least for the parasites detected in this study, even Hymenolepis nana can be directly transmitted.
   Thank you for this critique and we agree. We have, accordingly, toned down and replaced this sentence with the following, lines 79-81: "Many human intestinal parasites are also zoonotic and can be transmitted to or from other animals, some of which can also cause illness and occasional death in the animal, and as well as economic loss for the animal's owner [7–10]. "

6. Lines 76-82: this paragraph can be removed, unless information on infections among animals can be added.
   We agree and have removed the following sentence "Therefore, zoonotic IPIs are most appropriately assessed with a One Health approach, to understand the transmission and persistence of IPIs in humans, animals, and the environment, and to improve the health of humans and animals in endemic settings [8–10]." We replaced it with the following sentence, lines 77-81: "The majority of IPIs are transmitted via the faecal-oral route, either through faecally contaminated hands, food, water, vegetation, other surfaces, and aerosolized particles [2,4–6]. Many human intestinal parasites are also zoonotic and can be transmitted to or from other animals, some of which can also cause illness and occasional death in the animal, and as well as economic loss for the animal's owner [7–10]." We believe this is important background information for the reader on the transmission of IPIs.

7. Line 321: state the overall prevalence first; i.e. those infected with at least one parasite species.
   Thank you and we agree. Therefore, we have added the following, line (331): "The overall prevalence of intestinal parasitic infections (IPIs) was 35%"

8. Tables 5 and 6: modify the title to include the bivariate and multivariate analyses.
   We agree with this and have incorporated your suggestion in both tables accordingly.

9. Table 5: on positive case is missing. The total is 79 while it is 80 in Table 4.
   Thank you for your attention to detail. Out of the 366 children included, 366 had a parasitology report, while only 345 had a parasitology report with a corresponding questionnaire. Therefore, in Table 4, we represented the overall prevalence of IPIs in the study (N=366), while Table 5 and 6 showed the risk factor univariate and multivariate analyses, in which the 21 missing questionnaire participants were not included (N=345). This would make it appear as if 1 positive case is missing. These numbers are now also clearly indicated in the flow-chart in Figure S1.

10. Tables 5 and 6: remove the % sign from the results of 2nd column.
    We agree with this and have incorporated your suggestion in both tables accordingly.

11. Lines 380-384: avoid repeating results; focus on main findings only.
    Agree. We have removed repeated results from this section.

Reviewer #2: (No Response)

Reviewer #3: Minor revision Thank you for your feedback.

Summary and General Comments

Reviewer #1:

1. The study and findings are not novel.
   We respectfully disagree with the reviewer. Of course, risk factors for IPIs have been investigated in countless studies. However, as the risk factors vary on a regional scale, we feel that the study is novel in the sense that it reports data on a region where we lack data. In general studies including pastoralists are extremely rare. This will prove important for policy makers and to improve interventions focussing on populations which are often insufficiently covered by health care.

2. Anthropometric indices were calculated and results on the prevalence and associated factors of wasting, stunting and underweight were presented and discussed with few related supplementary tables were provided. However, these were not indicated among the objectives of the study. However, stunting, wasting and underweight should be added as variables or potential predictors of Giardia and Ascaris (tables 5 & 6). Otherwise, text and results about nutritional status should be removed from the manuscript.
   Thank you for this point. We removed the nutritional status prevalence estimates as well as univariate and multivariate supplementary tables and text in the results and discussion related to nutritional status measures (see also our response to reviewer #1 point 3).

3. Rationale of "Owns cattle" grouping and cut-off are unclear.
   Thank you for this critique. The grouping and cut offs for the categorical variable "owns cattle" were based on the median of the original numerical variable. We have added information on categorization of numerical variables in the statistical analysis section, lines 222-223, "Categorization of numerical variables, such as ownership of cattle, were based on the median.".

4. This is a small regional study among small population restricted to Adadle woreda of the Somali Region. Woreda is the district level and kebele is the smallest administrative unit of Ethiopia. Adadle woreda is one of 10 districts of Shabelle Zone of the Somali Region State of Ethiopia.
   Thank you for your feedback. We adapted the text to reflect the fact that we recruited in eight villages of this region.

Reviewer #2: (No Response)

Reviewer #3: The MS titled: Prevalence and associated risk factors of intestinal parasitic infections in young pastoralist and agro-pastoralist children in the Somali Region of Ethiopia is well written and the finding in this study might be useful to the parasitologists, public health practitioners and public in general. Though there are observation and question as highlighted in the MS. The observations are as follow

1. Line 25: Consider deleting young from the young children
   We agree with this and have incorporated your suggestion throughout the manuscript.

2. Line 67: As above
   We agree with this and have incorporated your suggestion throughout the manuscript.

3. Line 78: separate the lifecycle
   Thank you for this suggestion. We have removed this sentence, as another reviewer did not agree with the phrasing either.

4. Line 102: Consider: The study assessed the prevalence of intestinal.........
   Thank you for this suggestion and we agree. We have changed this sentence, which now reads as follows (see lines 101-104 in the clean, revised version): "The study assessed the prevalence of intestinal parasitic infections and associated risk factors in children in pastoralist and agro-pastoralist communities in the Adadle woreda (district) of the Shabelle zone of the Ethiopian Somali Regional State (ESRS)."

5. Line 115: Complete the: obtained from the parent
   Thank you and completed.

6. Line 126: Mention the annual rainfall too
   We agree with this and have incorporated your suggestion in the Methods, line 128 in the revised, clean version, " …. and a mean annual rainfall of 300 mm."

7. Line 193: Delete the highlighted and start with: The samples.......
   We agree with this and have incorporated your suggestion in the manuscript.

8. Line 323: Change infestation to infection
   We have changed this in the manuscript.

9. Line 326: How did you identify to species level the Entamoeba? State this in your material and method. But if the method used could only identify to genus level then just refer to Entamoeba spp. in your result and subsequent discussion
   Thank you for this question. We could not identify *Entamoebae* beyond the genus level, therefore instead of *E.Histolytica/Dispar*, we have replaced these with "*Entamoeba* spp." throughout the manuscript.

10. Line 330: You referred to detection of the helminth parasites. Was it the adult parasites you detected or their eggs? if it is their eggs, then refer to the eggs and not the parasite. Also, the word hookworms is wide, mention the hookworm eggs detected. In that section also, you did not define H. nana
    Thank you for this question. We used the Kato-Katz method; therefore we detected helminth eggs. We agree with your suggestion and have changed all mention of helminth parasite to helminth eggs throughout the manuscript. We have changed hookworm to "*Ancylostoma* spp./*Necator* spp. (hookworm)" throughout the manuscript and in all applicable tables (4-6). Thank you for pointing out that we did not define *H. nana.* We have changed the text accordingly.

11. Line 372-376: The first two statements contradict, hence stick with one
    Agree. The second sentence has been removed.

12. Line 380-384: The highlighted are results, delete. Also, you dwell so much on result than the discussion in discussion section
    Agree and thank you for this critique, we have removed direct mention of results here and throughout the discussion.

13. Line 459: due seasonal , should be changed to, due to seasonal
    Thank you and we have changed the text accordingly.

Comments to authors

1. **General comments**:
   a. The manuscript is well written. The study is also well designed. The objectives are also clear and addressed with appropriate parasitological and clinical methods. There are however some minor points to be addressed by authors.
   Thank you.

   b. Some local names (e.g., Kebele, Woreda etc) should be first defined and be italicized to make it clear to readers who do not know about administrative units in Ethiopia.
   We agree and thank you for this critique. We have done so throughout the manuscript for woreda (district) and kebele (ward).

2. **Title:**
   The title can be modified by omitting "young". It is good to say simply "Prevalence and………………. parasitic infections in pastoralist and agro-pastoralist children……………..”
   Agree and changed.

3. **Abstract**:
   a. Although the main focus of the study is about the prevalence of IPIs, it is also good to include the degree of wasting and stunting in the result section of the abstract.
   Since our sampling included selection of severely wasted and stunted children, we have removed all prevalence and risk factor estimates on nutritional status measures (wasting, stunting, and underweight) out of the manuscript. This was based on the critique of a reviewer, and we agreed with their critique. We thus did not include prevalences on nutrition-related disorders as they are likely over-estimated compared to the actual prevalence observed in the general population.

4. **Introduction:**
   In the last paragraph (page 5), study participants close interaction with animals (cattle/ goats/ camels) is emphasized. It is also good to describe their living condition and interactions among neighborhood, especially in raising children as this would increase the risk of transmission of IPIs.
   Thank you for pointing this out. We agree with this comment and have added the following in the Introduction, lines 96-97 of the clean revised version: "Communities are tight-knit and often blood related, often sharing childcare, water and food resources, and medicines."

5. **Selection criteria:**
   a. In line 151, it is mentioned that stunting and wasting were done according to WHO. Which protocol? Please show the specific year of publication by WHO (see line 151 and 173).
   Thank you for this question. We used the 2006 WHO protocol and have added in this information in the new manuscript, lines 153-154 in the clean, revised version: "…according to the 2006 WHO growth standards" and lines 177-178 in the clean, revised version: "… were calculated using the 2006 WHO growth standard tables"

6. **Stool samples:**
   a. In lines 206 and 207, it is indicated that children who tested positive were given anti-parasitic drugs. It is good to move this statement to "Ethical Consideration" part. Please mention the names of the drugs administered.
   Thank you for pointing this out. We agree with this comment and have moved this line to Ethical consideration, now lines 116-118 in the clean, revised version and added the drugs given: "If a

child was found to have an intestinal parasitic infection, the child's mother was given anti-parasitic medications (mebendazole, albendazole or metronidazole) to administer to her child."

7. **Statistical analysis:**
   a. Although it is common to use "bivariate" and "univariate" analysis interchangeably, it is preferred to use the latter (univariate) as it can be clearly distinguished from multivariate analysis. "Bivariate" looks as if it were you used two variables in the analysis. In logistic regression model build-up, it is recommended to say " uni- vs-multi". Use consistently this style throughout the manuscript.
   Thank you for pointing this out. We have changed bivariate to univariate throughout the manuscript, including all affected tables (table 5 and 6).

   b. Nothing is mentioned about cut-off point of p-values for purposeful selection of variables for multivariate logistic regression build-up (please see the article by Bursac et al., 2008; purposeful selection of variables…..)
   Thank you for this critique and we have added the information on how we selected our variables in the methods, lines 225-227 in the clean, revised version: "Variables in the multivariable model were pre-selected based on the published literature and complemented with variables associated in the univariable analysis ($p < 0.2$) [37]."

8. **Results**
   a. In table 1 and others, the age of the children was classified as "2-3", "3-4" and "4-5" years. I checked if there was double-counting. But, the numbers (frequency) are correct. It is a bit confusing about the overlap of age categories. So, make it clear by using legend under the first table or using month instead of year for estimation of the age of study participants.
   Thank you for your attention to detail and for pointing this out. We agree and have changed the age grouping to months (23-35 months, 36-47 months, 48-60 months), so there is no overlap and no confusion for the reader. This has been changed throughout the manuscript and in all applicable tables (Tables 1, 5, 6).

   b. In table 4, please use the lower case for first letters for specific names of the genus *Entamoeba*
   Thank you for spotting this error, which is now corrected.

   c. What do you mean by "positive association" (line 343 and others). In statistics, there is difference between "association" and "correlation." We say positive/negative for correlation, but not for association. What has been described here shows the association as it relates to your hypothesis/research question. Please avoid the word "positive" throughout the manuscript. I think you want to say "there is significant statistical association between *G. lamblia* infection and the risk factors (e.g., age, source of water, ownership of cattle).
   Thank you for this critique and we agree. We have changed the wording accordingly from previously: "Bivariate analysis revealed children aged 3 – 4 years old were positively associated with G. lamblia infection. Similarly, multivariate analysis for A. lumbricoides infection revealed a significant positive association with the age group 3 – 4 years, in comparison to 2 – 3 years and 4 – 5 years." to now, line 462 in the clean, revised version: "Children aged 36-47 months old were associated with *A. lumbricoides* infection." All other locations where this wording was used in the original submitted manuscript (lines 468-482 in the clean, revised version) were removed due to other comments by reviewers.

   d. Please re-write the statements describing the odds of the likelihood of events. "Sourcing drinking water……. had an adjusted odds ratio of………" In multivariate analysis, you have already done

an adjustment, so no need of describing as " had an adjusted odds ratio etc". Instead show it as "aOR or ACOR" in bracket with 95 % CI.
Thank you for this suggestion and we have changed the affected statements by replacing "adjusted" with "aOR".

9. **Discussion:**
   a. In line 377, " ….of which there are an estimated 20-30 million" is NOT clear. 20-30 million? Humans/ cattle? Please qualify this statement.
   Thank you for this critique and we agree. We have changed the wording accordingly to the following, lines 385-388 in the clean, revised version: "In addition, this study adds to the limited body of research on the health of pastoralist communities living in the ESRS and in the horn of Africa, the latter geographic zone encompassing an estimated 20-30 million humans living a pastoral lifestyle [26,27]."

   b. Line 380. IPIs prevalence is medium. Why? Overprotection of children not to play outside on ground ? or What? Please give plausible biological/behavioural reasons taking into account both children and parents behaviours/practices . "Outdoor "open field" defecation was quite common in the study area (99.4% in agro-pastoralist area vs 92.8% in pastoralist area).
   Thank you for the question. We have removed mention of medium (based on a previous critique) but added the following plausible reasons for this difference in prevalence between different study settings and groups in Ethiopia: Discussion, lines 391-400 in the clean, revised version: "This difference is likely due to the effect of seasonality on transmission and prevalence of IPIs, as well as on nutritional status, which is known to have synergistic interactions with IPIs. In other regions of Ethiopia, IPI prevalences in pre-school aged children varied (between 17% and 58%), with most studies conducted in non-pastoralist communities, at hospitals or schools and in wet or mountainous zones [6,11–17]. Pastoralists in nearby Chad were found to have a higher prevalence of IPIs (60%). Ecological differences and cultural differences such as behaviours around hygiene, animals, and food, and child rearing, weaning and outdoor play may all impact IPI prevalence in each study setting, making comparisons difficult."

   c.

   d. Did you compare with similar conditions in pastoralists in neighbouring countries (Somalia, Kenya etc if there are any reports in these countries).
   Thank you for this question. We searched for other studies on pastoralist children and IPIs in the region and only found 1 paper in Chad, which we cited earlier on in the manuscript, but have added in the following sentence in the discussion, lines 396-397 in the clean, revised version: "Pastoralists in nearby Chad were found to have a higher prevalence of IPIs (60%)." There were no comparable studies done in Kenya, Somalia, Eritrea, or other nearby countries, as pastoralists are a very understudied group of people.

   e. Higher odds of infection with *A. lumbricoides* in children aged 3-4 years. Why? You said that "specific behavioural trait." Please elaborate this statement. This age is usually the time at which children start to crawl and go outside. This might expose them to ova of *Ascaris* in soil. Please use an appropriate citation for elaborating this statement.
   Thank you for this question. We have added the following to the discussion, lines 466-469 in the clean, revised version: "As *A. lumbricoides* is transmitted via the faecal-oral route [2,6,9], age-specific changes such as initiation of exploratory behaviour outside of the home, playing with faeces-contaminated soil, and loss of immunologic protection from breastmilk following the weaning period may contribute to a heightened risk in the 36-47 month old group [16,44]."

   f. How are stunting and wasting compared with national report (if any in the specific age group or < five-year children)?

Thank you for this question. Since our sampling included selection of severely wasted and stunted children, we are not able to make any robust estimations of the stunting or wasting prevalence in this population. All nutritional status prevalences and risk factors have thus been removed from the manuscript.

10. Conclusions:

a. In line 380, it is shown that prevalence of IPIs to be "**medium**." But, you here concluded that "**high**" prevalence was observed (line 506). The two statements will not go together. What is your reference (WHO report/ national or….?) to say " medium" or "high"?
Thank you for this critique and we agree. As we were not able to find a reference, we have removed all mention of "medium" and "high" prevalence from the manuscript.
