# Supplementary material for: Prevalence and associated risk factors of intestinal parasitic infections among children in pastoralist and agro-pastoralist communities in the Adadle woreda of the Somali Regional State of Ethiopia
Source: PLoS Negl Trop Dis. 2023 Jul 3;17(7):e0011448. doi: 10.1371/journal.pntd.0011448 (PMC10348586; doi:10.1371/journal.pntd.0011448)
Supplement: Attachment — Submitted filename: PNTD-D-22-01533_reviewed.pdf [file pntd.0011448.s012.pdf]

PLOS Neglected Tropical Diseases

Prevalence and associated risk factors of intestinal parasitic infections in young pastoralist and agro-pastoralist children in the Somali Region of Ethiopia

--Manuscript Draft--

**Manuscript Number:**	PNTD-D-22-01533
**Full Title:**	Prevalence and associated risk factors of intestinal parasitic infections in young pastoralist and agro-pastoralist children in the Somali Region of Ethiopia
**Short Title:**	Parasites in the Somali Regional State of Ethiopia
**Article Type:**	Research Article
**Keywords:**	Ethiopian Somali Regional State, Ethiopia, children <5 years, intestinal parasitic infection, Giardia lamblia, Ascaris lumbricoides, pastoralist, One Health, WASH, livestock animals
**Abstract:**	Background: Intestinal parasitic infections (IPIs) can cause illness, morbidity and occasional mortality in young children. Agro-pastoralist and pastoralist children in the Somali Regional State of Ethiopia (ESRS) are especially at risk for IPIs, as access to safe water, sanitation, and health services is lacking. Little data on IPI prevalence and risk factors exists in this region. Methodology: We assessed the IPI prevalence and associated risk factors in the wet season from May-June 2021 in 366 children aged 2–5 years in four agro-pastoralist and four pastoralist kebeles in Adadle woreda of the Shebelle zone, ESRS. A detailed household questionnaire, anthropometric measurements, and stool samples were obtained from included children. Parasites were identified microscopically using Kato-Katz and direct smear methods. Risk factors were assessed using general estimating equation models accounting for clustering. Principal Findings: Protozoan parasite prevalence was 24.9%: 21.9% Giardia lamblia, and 3.0% Entamoebahistolytica/dispar. Intestinal helminth prevalence was 14.5%: 12.8% Ascaris lumbricoides, 1.4% hookworms, and 0.3% Hymenolepis nana. Giardia lamblia infection prevalence was higher when drinking water was sourced from the river (27%, aOR 10.1; 95%CI 3.59, 28.3) and collected rainwater (21%, aOR 8.54; 95%CI 2.23, 32.7), compared to tank trucks, springs, or boreholes (3.6%), when households owned cattle (22%, aOR 1.70; 95%CI 1.09, 2.64 for 1-5 cattle and 30%, aOR 2.51; 95%CI 1.39, 4.54 for 6+ cattle, compared to owning no cattle 18%) or chicken (No 21% vs. Yes 50%, aOR 3.38; 95%CI 1.71, 6.69). Ascaris lumbricoides infection prevalence was higher in children 3–4 years old compared to younger children (aOR 1.91; 95%CI 1.03, 3.54). Conclusions/Significance: Improving access to safe water and sanitation services in Adadle and employing a One Health approach to treat children and animals for parasitic infections are important to reduce IPIs in young children living in (agro-)pastoralist communities in Adadle and ESRS.
**Additional Information:**	
**Question**	**Response**
**Government Employee**<br><br>Are you or any of the contributing authors an employee of the United States government?<br><br>Manuscripts authored by one or more US Government employees are not copyrighted, but are licensed under a CC0 Public Domain Dedication, which allows unlimited distribution and reuse of the article for any lawful purpose. This is a	No - No authors are employees of the U.S. government.

Powered by Editorial Manager® and ProduXion Manager® from Aries Systems Corporation

legal requirement for US Government employees.<br><br>This will be typeset if the manuscript is accepted for publication.	
**Financial Disclosure**<br><br>Enter a financial disclosure statement that describes the sources of funding for the work included in this submission and the role the funder(s) played. This includes grants and any commercial funding of the work or authors.<br><br>This statement will be typeset if the manuscript is accepted for publication.<br><br>Review the submission guidelines and the instructions link below for detailed requirements and guidance.	This study received financial support from the Swiss Agency for Development and Cooperation (SDC) (AM, no. 7F-09057.01.02), the Nutricia Research Foundation (PV, no. 2019-20), the Forschungsfonds of the University of Basel (PV, Fellowship in 2020), the SNSF Return Grant (PV, no. P3P3PA_177877) and the Eccellenza Fellowship (PV, no. PCEFP3_194545). The funders had no role in study design, data collection and analysis, decision to publish, or preparation of the manuscript.
**Competing Interests**<br><br>On behalf of all authors, disclose any competing interests that could be perceived to bias this work.<br><br>This statement will be typeset if the manuscript is accepted for publication.<br><br>Review the instructions link below and PLOS NTDs' competing interests policy to determine what information must be disclosed at submission.	The authors declare that they have no conflict of interest.
**Data Availability**<br><br>Provide a **Data Availability Statement** in the box below. This statement should detail where the data used in this submission can be accessed. This statement will be typeset if the manuscript is accepted for publication.	All data can be found in the Supplementary Material of this article.

Powered by Editorial Manager® and ProduXion Manager® from Aries Systems Corporation

Before publication, authors are required to make all data underlying their findings fully available, without restriction. Review our PLOS Data Policy page for detailed information on this policy. Instructions for writing your Data Availability statement can be accessed via the Instructions link below.	

Manuscript Click here to access/download;Manuscript;Lankeretal_Parasite_mauscript_0

Prevalence and associated risk factors of intestinal parasitic infections in young pastoralist and agro-pastoralist children in the Somali Region of Ethiopia

Kayla C. Lanker[1,2¶], Abdifatah M. Muhummed[1,2,3¶], Guéladio Cissé[2,4], Jakob Zinsstag[1,2,3], Jan Hattendorf [1,2], Ramadan Budul Yusuf [3], Shamil Barsenga Hassen[3], Rea Tschopp[1,5], Pascale Vonaesch[6*]

[1] Human and Animal Health Unit, Swiss Tropical and Public Health Institute, Basel, Switzerland

[2] Faculty of Science, University of Basel, Basel, Switzerland

[3] Jigjiga University One Health Initiative, Jigjiga University, Jigjiga, Ethiopia

[4] Ecosystem Health Sciences Unit, Swiss Tropical and Public Health Institute, Basel, Switzerland

[5] One Health Unit, Armauer Hansen Research Institute, Addis Ababa, Ethiopia

[6] Department of Fundamental Microbiology, University of Lausanne, Lausanne, Switzerland

* Corresponding Author

Email: pascale.vonaesch@unil.ch (PV)

[¶]These authors contributed equally to this work.

Abstract

Background: Intestinal parasitic infections (IPIs) can cause illness, morbidity and occasional mortality in young children. Agro-pastoralist and pastoralist children in the Somali Regional State of Ethiopia (ESRS) are especially at risk for IPIs, as access to safe water, sanitation, and health services is lacking. Little data on IPI prevalence and risk factors exists in this region. **Methodology:** We assessed the IPI prevalence and associated risk factors in the wet season from May-June 2021 in 366 children aged 2–5 years in four agro-pastoralist and four pastoralist kebeles in Adadle woreda of the Shebelle zone, ESRS. A detailed household questionnaire, anthropometric measurements, and stool samples were obtained from included children. Parasites were identified microscopically using Kato-Katz and direct smear methods. Risk factors were assessed using general estimating equation models accounting for clustering. **Principal Findings:** Protozoan parasite prevalence was 24.9%: 21.9% *Giardia lamblia*, and 3.0% *Entamoeba histolytica/dispar*. Intestinal helminth prevalence was 14.5%: 12.8% *Ascaris lumbricoides*, 1.4% hookworms, and 0.3% *Hymenolepis nana*. *Giardia lamblia* infection prevalence was higher when drinking water was sourced from the river (27%, aOR 10.1; 95%CI 3.59, 28.3) and collected rainwater (21%, aOR 8.54; 95%CI 2.23, 32.7), compared to tank trucks, springs, or boreholes (3.6%), when households owned cattle (22%, aOR 1.70; 95%CI 1.09, 2.64 for 1-5 cattle and 30%, aOR 2.51; 95%CI 1.39, 4.54 for 6+ cattle, compared to owning no cattle 18%) or chicken (No 21% vs. Yes 50%, aOR 3.38; 95%CI 1.71, 6.69). *Ascaris lumbricoides* infection prevalence was higher in children 3–4 years old compared to younger children (aOR 1.91; 95%CI 1.03, 3.54). **Conclusions/Significance:** Improving access to safe water and sanitation services in Adadle and employing a One Health approach to treat children and animals for parasitic infections are important to reduce IPIs in young children living in (agro-)pastoralist communities in Adadle and ESRS.

Author summary

Intestinal parasitic infections remain a silent threat to the health and life-trajectories of children living in areas with inadequate access to clean water, proper sanitation and hygiene facilities, including in the Somali region of Ethiopia. A large majority in this region live as pastoralists (semi-mobile animal herders), in close contact with their animals and nature, at risk for climate-related threats like drought and flooding, and at risk for infectious agents like intestinal parasites. In this study, we assessed the prevalence of intestinal parasitic infections in young pastoralist children in this region, and the individual and household-level factors associated with these infections. We found that locally collected water, along with ownership of cows and chickens increased the risk for having an intestinal parasitic infection with *Giardia lamblia*, which can cause diarrhea and is transmitted through water, food, and soil that have been contaminated by the feces of infected humans and animals. If access to clean water and sanitation facilities is not improved, these infections remain recurrent in these communities and their animals, continually affecting the health of children. This study is one of few involving pastoralists in this region, hopefully lending guidance to regional public health policies.

Introduction

Intestinal parasitic infections (IPIs) remain one of the most common infectious diseases in humans and animals globally with a high burden of disease and occasional mortality [1,2]. Young children are especially at risk for IPIs due to changes in nutrition, age-specific behaviours around playing and hygiene, and developing immune systems [1]. Additional risk factors include lack of access to clean water, inadequate sanitation and hygiene, lack of knowledge on transmission pathways and lack of epidemiological surveillance [2]. While the

morbidity of specific IPIs varies, children chronically infected can develop iron (anemia), vitamin A and other micronutrient deficiencies, become undernourished, experience prolonged diarrheal episodes (dysentery), develop cognitive delays, become stunted, and may even die as a result of acute or chronic infection [1,3].

The majority of IPIs are transmitted via the faecal-oral route, either through faecally contaminated hands, food, water, vegetation, other surfaces, and aerosolized particles [2,4–6]. Most human intestinal parasites require an intermediate animal host to complete their lifecycle, often causing illness and death in the animal, and as well as economic loss for the animal's owner [7]. Therefore, zoonotic IPIs are most appropriately assessed with a One Health approach, to understand the transmission and persistence of IPIs in humans, animals, and the environment, and to improve the health of humans and animals in endemic settings [8–10].

In Ethiopia, IPIs in children have been demonstrated at high prevalences (between 42% and 53%) in children under five years of age [6,11–17] and school-aged children [18–23], from which associated risk factors were unsafe or contaminated drinking water [6,13], no sanitation facilities and open defecation [6,11,12], poor hygiene and knowledge on transmission [6,11,13], living in crowded homes [12], eating raw vegetables [6,12,17], and coming into contact with contaminated soils [6,17]. However, a 2019 systematic review found that in several regions of Ethiopia, including the Ethiopian Somali Regional State (ESRS), no prevalence estimates of IPIs in young children were published or available [24]. Indeed, to date, there is a single study assessing undernutrition and IPI prevalence in young pastoralist children in the ESRS during the drought season 2019 [25]. There is no data on prevalence in the wet season nor information on associated risk factors of IPIs, leaving a knowledge gap regarding IPIs in pastoralist communities in the ESRS.

Pastoralist and agro-pastoralist communities make up the majority of the total population of the ESRS [26,27]. They rely mainly on their animals for food (milk and meat) and livelihood, are semi-mobile and lack access to safe water and sanitation as well as health services [25,28]. As pastoralists live in close connection and proximity to their animals and their environment year-round, it is expected that sharing of many microbial species, including intestinal parasites, is high. Here, we aimed to assess the factors associated with intestinal parasitic infection in young children in pastoralist and agro-pastoralist communities in the Adadle woreda (district) of the Ethiopian Somali Regional State (ESRS). The study is part of the Jigjiga One Health Initiative to improve the health of humans, animals, and their environments among pastoralist communities in the ESRS, with a specific focus on improving the health of young children in the ESRS.

Methods

Ethics statement

Ethics approval for this study was obtained from the Review Committee of the University of Jigjiga in Ethiopia (JJU-RERC030/2020) and the Review Committee of Armauer Hansen Research Institute in Addis Ababa, Ethiopia (AF-10-015), and from the Swiss Ethics Committee of Northwest and Central Switzerland (Ethikkommision Nordwest- und Zentralschweiz; REQ-2020-00608). Oral or written consent was obtained by the parents of all participating children before study enrolment. Data was recorded on the Open Data Kit and stored on a secured server at the Swiss TPH in Basel. All identifying information was kept with the local study team in Ethiopia and is securely stored in a closed cupboard.

Study design

Study Area

The study is a cross-sectional study in the Adadle Woreda in the Shebelle zone of the Somali Regional State of Ethiopia and was carried out from May 2021 until June 2021 during the wet season, known locally as *gu-'ga*. Adadle is located 17 km from the city of Gode in the lowlands of the Wabi Shabelle River subbasin and experiences a mean annual temperature of 32˚C. The altitude is 300-500 meters above sea-level and 80% of the land is flat, while 20% is undulated. The pastoralist and agro-pastoralist communities in ESRS and Adadle rely mainly on their animals for food and livelihood and live largely outside of modern systems.

Sample Size

The sample size was determined based on several components, as this study was part of a cooperative project further studying antimicrobial resistance and the microbiome in the same study population. Based on a previous study in children aged 2 - 5 years in this region, we expected the prevalence of parasitic infection by either helminth and/or protozoan parasites to be 42% [25]. As we expected clustering on the community (kebele) level, the intra-cluster correlation coefficient was assumed to be 0.15. We chose a 95% confidence interval, power of 80%, alpha of 0.05, and a margin of error of 10%. Based on these factors and to achieve the necessary sample size for all aims of the larger research project, we calculated a sample size of 360 eligible children, 180 each in pastoralist and agro-pastoralist communities.

Selection Criteria

Stakeholders (community leaders and health officials) in the ESRS Adadle woreda were approached at each level (regional, kebele, sub-kebele), and invited to help determine which

kebeles in Adadle woreda could be included in the present study. From the included kebeles, four pastoralist (Malkasalah, Todob, Harsug, Kulmis) and four agro-pastoralist (Bursaredo, Gabal, Higlo, Boholhagare) kebeles were randomly selected (**Fig 1**). A pre-enrolment screening was carried out at the local health centre in each kebele, for all children aged 2 - 5 years. Medication and hygienic supplies were given as incentive to each household to attend the pre-screening, regardless of further participation in the study. All children were screened for stunting (height for age) and wasting (mid-upper arm circumference for height; MUAC), according to the WHO. All screened children who were severely wasted based on their weight for height z-score (WHZ < -2) or severely stunted based on their height for age z-score (HAZ < -2) were automatically included in the study. Screened children presenting as non-stunted and/or non-wasted were randomly selected (random number generation) from an excel file containing all pre-screened households/children in the community, until the maximum sample size was reached for each community and the overall study. Children were excluded if they were older than 5 years, younger than 2 years and/or had taken antibiotics in the last 14 days.

Anthropometric measurements

Anthropometric measurements of height, weight, and mid-upper arm circumference (MUAC) were measured at least twice, or until the measurements were within 1 cm, 100 g, or 1mm of one another, respectively. The measurements were then averaged for each child. Height was measured by having the child stand up straight against a WHO-standard wooden measuring board set on flat ground. Attention was given that the child was looking forward, had relaxed shoulders, straight legs, no shoes on, and arms at their side. Weight was measured by having the child stand alone in the middle of a WHO-standard scale without shoes, and with light clothing. If the child was not willing or able to be weighed alone, then the mother was first weighed alone and then weighed again with the child in her arms. The final weight of the child

was therefore the weight of the mother alone subtracted from the weight of mother and child together. The scale was calibrated daily against known weights to ensure accuracy on all study days. The mid-upper arm circumference (MUAC) was measured using a WHO-standard tape measurer. In the pre-screening, z-scores for height-for-age (HAZ; stunting) and weight-for-height (WHZ; wasting) were calculated using WHO anthropometric tables. Following the end of data collection, HAZ, WAZ, and WHZ were recalculated using the R package ‘zscorer’ [29]. Stunting and wasting were defined as HAZ and WHZ z-scores < -2, while acute malnutrition (low MUAC) was defined by MUAC < 12.5 cm.

Data collection

A detailed questionnaire using the Open Data Kit (ODK) platform [30] was administered in the local language by field workers to the mother of the child. The questionnaire was divided into several sections: (1) child anthropometric measures, current health and health history of child (2) household characteristics and assets, and type and number of household animals, (3) WASH behaviours of child and household, (4) a nutritional survey of the child, and finally (5) breastfeeding and birth history of mother-child. Birth records often do not exist in pastoralist communities; therefore, age in months was estimated based on group discussions with the child’s mother and other familial and community members to determine seasonal (floods, month of year, drought) and festive events (Ramadan, other religious events) that occurred before or after the birth of the child. Geo-reference points were collected for most households sampled for spatial visualizations.

Stool sample collection and parasitological analyses

Stool samples were collected by the mothers in sterile specimen cups after detailed instruction by a trained field worker in the local language. Upon retrieval of the stool samples, the samples were immediately transferred to a cold box containing ice and aliquoted. All parasitology analyses were performed in the field in each kebele health clinic by trained parasitologists from Jigjiga University. The aliquot for parasitology was divided into four portions. One portion was used for the quantification of helminth ova using the thick-smear Kato-Katz detection method [31] under a microscope at 10x and 40x magnification, which allows for the detection of common helminth parasites such as hookworms, *Hymenolepis nana*, *Trichuris trichiura*, *Ascaris lumbricoides*, and *Schistosoma mansoni*. Another two portions were used for the detection of protozoan cysts and trophozoites using the direct smear method in duplicate. Briefly, a drop of saline and a drop of Lugol's iodine were each placed on one piece of a single sterile glass slide, then using a cotton swab, a small amount of stool sample was smeared onto each drop, covered with a glass slip and inspected under a microscope. The final portion of stool was fixed and stored in SAF (sodium acetate–acetic acid- formalin) at 4˚C as a backup [32]. The stool consistency of each stool sample was determined by a trained parasitologists. If the parasitology analysis showed a positive result, the child's mother was given anti-parasitic medications to administer to her child.

Statistical Analysis

All statistical analyses were performed in R Statistical software version 4.0.4 [33]. Tables were generated using the gtsummary package [34]. Bivariable and multivariable analysis were carried out using the general estimating equation logistic regression model for binary outcomes using the geepack package [35], accounting for clustering in the data and estimates parameters

on a population level. Spatial visualizations were performed on QGIS3 [36] and public geographic data was downloaded from open source Humanitarian Data Exchange (HDX) [37].

Results

A total of 366 children aged between two to five years from 270 households spread between eight kebeles in the Adadle Woreda, Shebelle Zone of the Ethiopian Somali Regional State (ESRS) were included in the present study, of which 184 children were from pastoralist and 182 from agro-pastoralist communities. For all enrolled children, parasitology reports were documented (N=366). However, for twenty-one children, a questionnaire was not recovered, due to logistical issues with the software and field activities. Therefore, 345 children had a complete questionnaire and parasitology report (**S1 Fig**). By applying household-level data from completed questionnaires of siblings in the same household, 13 of the 21 children with a missing questionnaire were able to be recovered for the household-level descriptive and bivariate analyses (N=358; **S1 Fig**).

Participant Characteristics

Participant characteristics are summarized in **Table 1**. The age and sex of enrolled children were distributed evenly, including between pastoralist and agro-pastoralist communities. Only 10.9% of children had a complete vaccination at the time of sampling. Only 44.3% of children were ever breastfed, while even fewer (8.4%) were exclusively breastfed (EBF) for the first 6 months. Of those who were breastfed, the median duration of breastfeeding was 12 months (Range: 2 - 28 months). Supplementary milk from household animals (cows, goats, camels) was given in the first 6 months of life to 57.5% of children, while 21.7% received supplementary milk after 6 months, and 20.8% were never given supplementary milk prior to weaning. Supplementary animal milk was given as early as 1 month and as late as 28 months, with the median start time being at 1 month old. Supplementary foods (mostly soft staple foods like porridge, rice, potatoes, and injera) were started in 23.9% of children in the first 6 months

of life, while the rest (76.1%) received supplementary food after 6 months of age and was given as early as 1 month but as late as 40 months, with a median start time at 6 months.

Table 1. Characteristics of (agro-) pastoralist children aged 2 to 5 years living in Adadle Woreda, Somali region, Ethiopia.

Characteristic	N = 345[1]
**Age**	
2 - 3 years	120 (34.8%)
3 - 4 years	106 (30.7%)
4 - 5 years	119 (34.5%)
**Sex**	
Female	169 (49.0%)
Male	176 (51.0%)
**Vaccination status**	
Complete vaccination	37 (10.9%)
Incomplete vaccination	301 (89.1%)
**Was child breastfed (Yes)**	153 (44.3%)
**EBF for first six months (Yes)**	29 (8.4%)
**Complementary milk starting**	
Before 6 months	196 (57.5%)
After 6 months	74 (21.7%)
Never gave other milk	71 (20.8%)
**Complementary food starting**	
Before 6 months	80 (23.9%)
After 6 months	255 (76.1%)

EBF, Exclusive breastfeeding; Complementary food included mostly soft staple foods cooked in milk. Overall statistics are shown, as group differences were minimal. Data collected in the wet season 2021.

[1]n (%)

Height, weight, and mid-upper arm circumference (MUAC) were similarly distributed between pastoralist and agro-pastoralist children (**Table 2**). The overall stunting (HAZ < -2), wasting (WHZ < -2), underweight (WAZ < -2) and low MUAC (MUAC <12.5 cm) were 14%, 30%, 17%, and 2.3%, respectively. Only 5% of children were both wasted and stunted. Multivariate analysis revealed a significant positive association of wasting status with male children (male

38% vs. female 21%; aOR 2.21; 95% CI 1.65, 2.95) and children aged 3 - 4 (28%; aOR 1.57; 95% CI 1.10, 2.23) or 4 - 5 years (38%; aOR 2.12; 95% CI 1.50, 3.00) versus children aged 2 – 3 years (22%), while children with *Giardia lamblia* infection had a decreased odds of being wasted (yes 22% vs. no 32%; aOR 0.58; 95% CI 0.41, 0.83; **S1 Table**). For stunting status, male children had an increased odds of being stunted (male 16% vs. female 11%; aOR 1.49; 95% CI 1.30, 1.71; **S2 Table**). Underweight status had an increased odds if the child was male (male 24% vs. female 9.5%; aOR 2.89; 95% CI 1.74, 4.77) and a decreased odds for pastoralist children (pastoralist 11% vs. agro-pastoralist 23%; aOR 0.39; 95% CI 0.20, 0.76; **S3 Table**). Low MUAC could not be analysed in multivariate analysis due to the low prevalence.

Table 2. Anthropometric characteristics of agro-pastoralist and pastoralist children aged 2 - 5 years living in Adadle woreda, Somali region, Ethiopia.

Characteristic	Agro-pastoralist N = 177[1]	Pastoralist N = 181[1]	Overall N = 358[1]
**Height (cm)**	96 (90, 102)	98 (90, 105)	97 (90, 104)
**Weight (kg)**	12.60 (11.38, 13.90)	13.30 (11.60, 15.00)	12.80 (11.40, 14.50)
**HAZ**	-0.37 (-1.45, 0.41)	0.02 (-0.97, 0.78)	-0.18 (-1.21, 0.59)
**Stunting (Yes)**	26 (15%)	21 (12%)	47 (14%)
**WHZ**	-1.55 (-2.18, -0.98)	-1.33 (-2.03, -0.65)	-1.41 (-2.15, -0.82)
**Wasting (Yes)**	53 (32%)	49 (28%)	102 (30%)
**WAZ**	-1.34 (-1.95, -0.72)	-0.83 (-1.62, -0.22)	-1.06 (-1.78, -0.40)
**Underweight (Yes)**	39 (23%)	19 (11%)	58 (17%)
**MUAC (cm)**	14.00 (13.50, 14.72)	14.20 (13.60, 14.90)	14.10 (13.50, 14.90)
**Low MUAC (Yes)**	5 (3.0%)	3 (1.7%)	8 (2.3%)

HAZ, height for age z-score; Stunting (Yes): HAZ < -2 SD; WHZ, weight for height z-score; Wasting (Yes): WHZ < -2 SD; WAZ, weight for age z-score; Underweight (Yes); WAZ < -2 SD; MUAC, mid-upper arm circumference; Low MUAC (Yes): MUAC <12.5 cm; SD: standard deviation.

[1]Median (IQR); n (%)

Household and Kebele Characteristics

The included agro-pastoralist kebeles all reside along or near the Shebelle River (**Fig 1**); two kebeles (7: Higlo; 8: Boholhagare) lived along the Shebelle River and were approximately 16 km downstream from Gode town, one kebele (6: Bursaredo) was near an agricultural zone 20 – 40 km from Gode banking the Shebelle River, and the last kebele (5: Gabal) banked the Shebelle River 50 – 70 km upstream of Gode town. The included pastoralist kebeles (1: Todob; 2: Kulmis; 3: Malkasalah; 4: Harsug) did not reside near any rivers, but did flank road systems, and were between 48 - 75 km from Gode town (**Fig 1**). Households (regardless of if pastoralist or agro-pastoralist) spoke exclusively the Somali language (100%), were of the Muslim faith (100%), were majority illiterate (head of household, 74.0%; mother of child, 75.1%), and a majority owned a mobile phone (60.6%) (**S4 Table**).

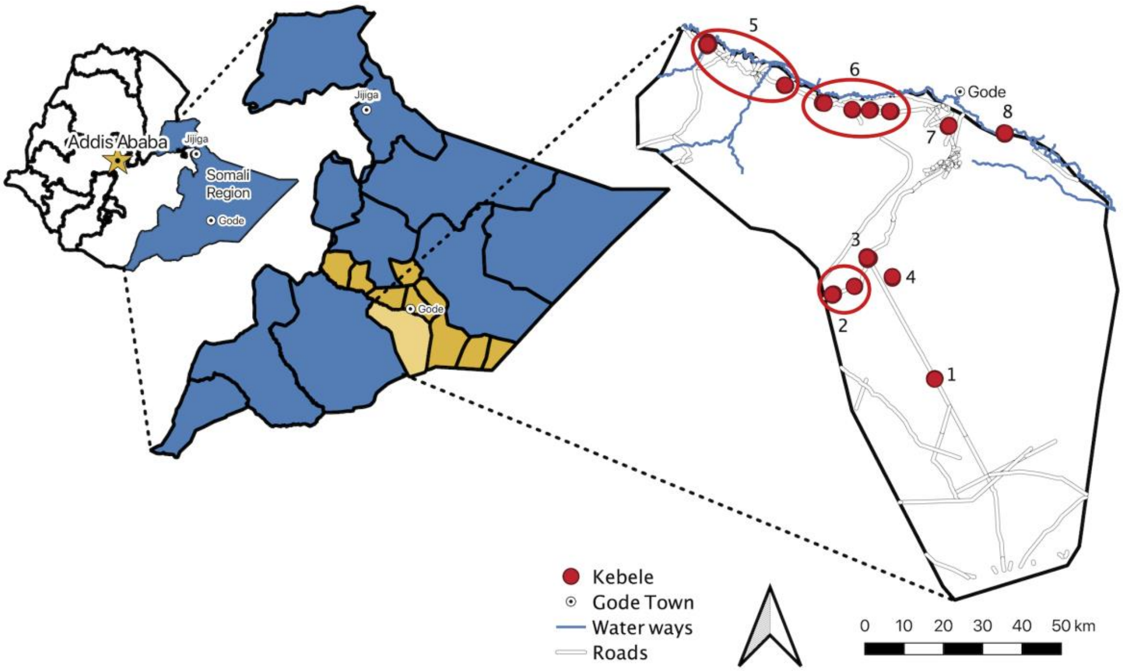

Fig 1. Map showing included kebeles in Adadle woreda in the Shebelle zone of the Somali regional state, Ethiopia.

Kebele locations are based on geopoints collected during the study. Kebeles 1 – 4 are pastoralist communities (1: Todob; 2: Kulmis; 3: Malkasalah; 4: Harsug) and kebeles 5 – 8 are agro-pastoralist communities (5: Gabal; 6: Bursaredo; 7: Higlo; 8: Boholhagare).

Household water, sanitation and hygiene (WASH) characteristics showed some differences between pastoralist and agro-pastoralist communities and are summarized in **Table 3**. The majority (85.3%) of agro-pastoralist communities sourced their water from the Shebelle River**,** while the majority (88.4%) of pastoralist households sourced their water from rain collected in open shallow wells or birkads. Only a small minority sourced their water from a borehole, tank truck or natural spring (agro-pastoralist: 5.6%, pastoralist: 9.9%). More agro-pastoralist (44.1%) than pastoralist (13.3%) treated their water, through chlorination. Agro-pastoralists almost exclusively practiced open defecation (99.4%), while pastoralists practiced open defecation 92.8% of the time and 7.2% indicated they used a type of pit latrine. Toilet sharing, meaning the sharing of a latrine or defecation spot, was shared by a minority in both agro-pastoralist (11.3%) and pastoralist (5.5%) communities. Waste disposal was similar between agro-pastoralists and pastoralists; the majority dumped their waste (81.3%), either in their compound, river or in the open, while few burned their waste (15.1%). Household ownership of soap was also quite low, with 57.1% indicating they do not usually have soap in the house. Finally, hygienic characteristics of the households were similar, with most mothers reporting that they washed their child’s hands with water only (93.7%), and few with water and soap (6.3%).

Table 3. Household WASH characteristics of agro-pastoralist and pastoralist children aged 2 - 5 years living in Adadle woreda, Somali region, Ethiopia.

	Characteristic	Agro-pastoralist N = 177[1]	Pastoralist N = 181[1]	Overall N = 358[1]
WATER	**Source of drinking water**			
	Borehole/Spring/Tank truck	10 (5.6%)	18 (9.9%)	28 (7.8%)
	Rainwater in Birkads	16 (9.0%)	160 (88.4%)	176 (49.2%)
	River water	151 (85.3%)	3 (1.7%)	154 (43.0%)
	**Treatment of water (Yes)**	78 (44.1%)	24 (13.3%)	102 (28.5%)
SANITATION	**Toilet type**			
	Pit latrine	1 (0.6%)	13 (7.2%)	14 (3.9%)
	Outdoor	176 (99.4%)	168 (92.8%)	344 (96.1%)
	**Shared toilet (Yes)**	20 (11.3%)	10 (5.5%)	30 (8.4%)
	**Waste disposal**			
	Burned	26 (15.5%)	28 (15.8%)	54 (15.7%)
	Dumped	142 (84.5%)	149 (84.2%)	291 (84.3%)
HYGIENE	**Household owns soap**			
	Yes	36 (20.5%)	15 (8.3%)	51 (14.3%)
	Sometimes	41 (23.3%)	61 (33.7%)	102 (28.6%)
	No	99 (56.2%)	105 (58.0%)	204 (57.1%)
	**Child hand washing method**			
	With water	146 (90.1%)	168 (97.1%)	314 (93.7%)
	With water and soap	16 (9.9%)	5 (2.9%)	21 (6.3%)

Birkads: open shallow wells or ditches; Treatment of water was mostly through chlorination.

[1]n (%)

Agro-pastoralists and pastoralists in the ESRS depend greatly on livestock animals as a means of food and livelihood. Household ownership of domestic animals, which includes in the present study cattle, camel, goats, sheep, donkeys, and chickens, varied in quantity between the agro-pastoralist and pastoralist communities (**Fig 2; S5 Table).** Agro-pastoralists owned more cattle, sheep, donkeys, and chickens, while pastoralists owned more goats and camels

and no chickens. Livestock animal ownership ranged from zero to 149 animals, with a median of 26 animals, and more animals were kept inside the house or compound of pastoralist households (73.5%) than agro-pastoralist households (58.8%) (**S5 Table**).

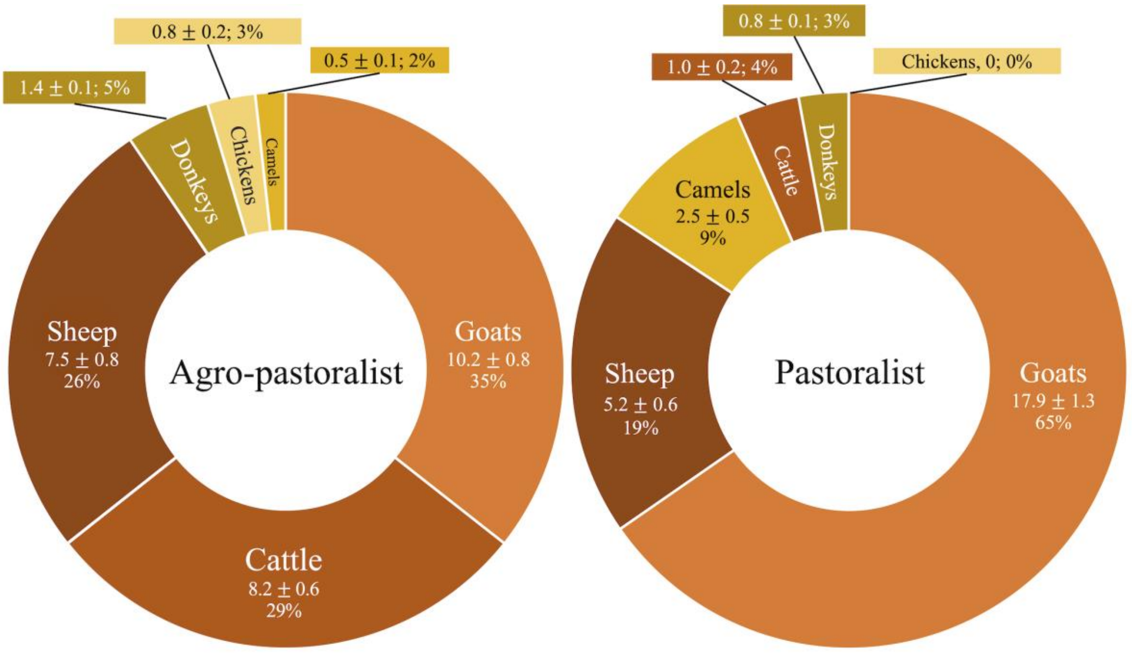

Fig 2. Average household animal herd makeup of agro-pastoralist and pastoralist children aged 2 - 5 years of age living in Adadle woreda, Somali region, Ethiopia. Numbers are the mean ± the standard error of the mean and mean percentage of each type of animal owned per participant per group (pastoralist or agro-pastoralist).

Intestinal parasitic infection prevalence

The overall prevalence of intestinal parasitic infections (IPIs) was 30.6% for a single parasite infection and 4.4% for poly-parasitic infections (coinfection with protozoan and helminth parasite). The infestation pattern is summarized in **Table 4**. Intestinal protozoan infections were detected in 24.9% of children, while intestinal helminth infections were detected in 14.5% of children. The prevalence of protozoan parasites was 21.9% *Giardia lamblia* and 3.0%

Entamoeba histolytica/*Entamoeba dispar*. Of the *G. lamblia* infections detected, 65% were in the cystic stage and 35% in the trophozoite stage. *G. lamblia* prevalence was slightly higher in agro-pastoralist communities (24.2%) compared with pastoralist communities (19.6%), although this difference was not significant in bivariate or multivariate analyses (**Table 5**). The overall prevalence of helminth parasites was 12.8% *Ascaris lumbricoides*, 1.4% Hookworm, and 0.3% *H. nana*. Both *A. lumbricoides* and hookworm had a slightly higher prevalence in agro-pastoralist communities compared with pastoralist communities, although this difference was not significant in bivariate or multivariate analyses (**Table 6**). Poly-parasitic infections (4.4%) were all found to be a co-infection of a protozoan with a helminth species, of which 25% (4/16) were *E. histolytica*/*dispar* with the helminth *A. lumbricoides* and 75% (12/16) *G. lamblia* with a helminth (*A. lumbricoides* 9/16; *H. nana* 1/16; Hookworm 2/16).

Table 4. Prevalence of intestinal parasitic infections during the 2021 wet season in agro-pastoralist and pastoralist children aged 2 - 5 years living in Adadle woreda, Somali region, Ethiopia.

**Characteristic**	**Agro-pastoralist N = 182[1]**	**Pastoralist N = 184[1]**	**Overall N = 366[1]**
**Overall**			
Parasite free	115 (63.2)	123 (66.8)	238 (65.0)
Single parasite	57 (31.3)	55 (29.9)	112 (30.6)
Poly-parasite	10 (5.5)	6 (3.3)	16 (4.4)
**Protozoa**			
Giardia lamblia	44 (24.2)	36 (19.6)	80 (21.9)
Entamoeba Histolytica/Dispar	3 (1.6)	8 (4.3)	11 (3.0)
**Helminths**			
Ascaris lumbricoides	26 (14.3)	21 (11.4)	47 (12.8)
Hookworm	4 (2.2)	1 (0.5)	5 (1.4)
Hymenolepis nana	0 (0.0)	1 (0.5)	1 (0.3)

[1]n (%)

Factors associated with *Giardia lamblia* infection

In bivariate analysis, *G. lamblia* infection was positively associated with children aged 3-4 years (compared to 2 - 3 years), source of drinking water, ownership of cattle and ownership of chickens. In multivariate analysis, *G. lamblia* infection remained associated with water source, and ownership of cattle and chicken (**Table 5**). Sourcing drinking water from rainwater had an adjusted odds of 8.54 (95% CI 2.23, 32.7; p-value = 0.002), while sourcing from river water had an adjusted odds of 10.1 (95% CI 3.59, 28.3; p-value < 0.001), when compared to sourcing water from a spring, tank truck, or borehole. Household ownership of cattle revealed an adjusted odds of 1.70 (95% CI 1.09, 2.64; p-value = 0.019) when owning 1 - 5 cattle, and an adjusted odds of 2.51 (95% CI 1.39, 4.54; p-value = 0.002) when owning more than 6 cattle, compared with owning no cattle. Similarly, owning chickens had an adjusted odds of infection of 3.38 (95% CI 1.71, 6.69; p-value < 0.001), compared to owning no chickens. All further variables included in multivariate analysis were non-significant. Bivariate and multivariate was not possible for *E. histolytica*/*dispar*, due to low positive numbers.

Table 5. Multivariate Analysis of *Giardia lamblia* in agro-pastoralist and pastoralist children aged 2 - 5 years living in Adadle woreda, Somali region, Ethiopia.

	**Bivariate, N = 358**		**Multivariate, N = 345**
**Characteristic**	**n/N (% Positive)**	**OR (95% CI)[1]**	**aOR (95% CI)[1]**
**Group**			
Agro-pastoralist	43 / 177 (24%)	—	—
Pastoralist	36 / 181 (20%)	0.77 (0.36, 1.67)	1.86 (0.48, 7.26)
**Sex[2]**			
Female	38 / 169 (22%)	—	—
Male	38 / 176 (22%)	0.95 (0.62, 1.45)	0.99 (0.63, 1.55)
**Age[2]**			
2 - 3 years	21 / 120 (18%)	—	—

	Bivariate, N = 358		Multivariate, N = 345
**Characteristic**	**n/N (% Positive)**	**OR (95% CI)[1]**	**aOR (95% CI)[1]**
3 - 4 years	30 / 106 (28%)	1.86 (1.10, 3.14)*	1.69 (0.93, 3.05)
4 - 5 years	25 / 119 (21%)	1.25 (0.84, 1.88)	1.18 (0.74, 1.87)
**Drinking water source**			
Borehole/Spring/Tank truck	1 / 28 (3.6%)	—	—
Rainwater	37 / 176 (21%)	7.19 (2.42, 21.3)***	8.54 (2.23, 32.7)**
River water	41 / 154 (27%)	9.80 (2.72, 35.3)***	10.1 (3.59, 28.3)***
**Owns cattle**			
No	24 / 137 (18%)	—	—
1-5 cattle	30 / 137 (22%)	1.32 (0.68, 2.55)	1.70 (1.09, 2.64)*
6+ cattle	25 / 84 (30%)	2.00 (1.29, 3.09)**	2.51 (1.39, 4.54)**
**Owns chickens**			
No	70 / 340 (21%)	—	—
Yes	9 / 18 (50%)	3.86 (2.52, 5.91)***	3.38 (1.71, 6.69)***

OR = Odds Ratio; aOR = Adjusted Odds Ratio; Bivariate and multivariate analyses performed using a general estimating equation logistic regression model for binary outcomes.

[1]*p<0.05; **p<0.01; ***p<0.001; [2]N = 345 for bivariate analysis

Factors associated with *Ascaris lumbricoides* infection

In bivariate analysis, *Ascaris lumbricoides* was positively associated with children aged 3 - 4 years and with owning more than 6 cattle (**Table 6**). In multivariate analysis, *A. lumbricoides* infection remained positively associated with children aged 3 – 4 years (aOR 1.91; 95% CI 1.03, 3.54; p-value = 0.041) compared to children aged 2 – 3 years. All further variables included in multivariate analysis were non-significant. Bivariate and multivariate was not possible for the other helminth parasites due to low positive numbers.

Table 6. Multivariate Analysis of *Ascaris lumbricoides* in agro-pastoralist and pastoralist children aged 2 - 5 years living in Adadle woreda, Somali region, Ethiopia.

	Bivariate, N = 358		Multivariate, N = 345
**Characteristic**	**n/N (% Positive)**	**OR (95% CI)[1]**	**aOR (95% CI)[1]**
**Group**			
Agro-pastoralist	26 / 177 (15%)	—	—
Pastoralist	21 / 181 (12%)	0.76 (0.39, 1.50)	1.08 (0.52, 2.23)
**Sex[2]**			
Female	21 / 169 (12%)	—	—
Male	25 / 176 (14%)	1.17 (0.69, 1.97)	1.26 (0.70, 2.25)
**Age[2]**			
2 - 3 years	14 / 120 (12%)	—	—
3 - 4 years	22 / 106 (21%)	1.98 (1.09, 3.60)*	1.91 (1.03, 3.54)*
4 - 5 years	10 / 119 (8.4%)	0.69 (0.23, 2.10)	0.70 (0.23, 2.13)
**Shared toilet**			
No	42 / 328 (13%)	—	—
Yes	5 / 30 (17%)	1.36 (0.53, 3.50)	1.29 (0.38, 4.42)
**Household owns soap**			
Yes	22 / 153 (14%)	—	—
No	25 / 204 (12%)	0.83 (0.40, 1.75)	0.85 (0.40, 1.78)
**Owns cattle**			
No	14 / 137 (10%)	—	—
1-5 cattle	17 / 137 (12%)	1.24 (0.70, 2.21)	1.38 (0.80, 2.40)
6+ cattle	16 / 84 (19%)	2.07 (1.09, 3.93)*	1.98 (0.92, 4.24)

OR = Odds Ratio; aOR = Adjusted Odds Ratio; Bivariate and multivariate analyses performed using a general estimating equation logistic regression model for binary outcomes.

[1]*p<0.05; **p<0.01; ***p<0.001; [2]N = 345 for bivariate analysis

Discussion

To our knowledge, this is the first study assessing the impact of livestock animal keeping and WASH characteristics on the risk of intestinal parasitic infections (IPIs) in children 2 - 5 years of age living in pastoralist and agro-pastoralist communities in the ESRS. It is the second study

in the region to demonstrate medium-high levels of certain IPIs, which are of public health importance. Additionally, this study adds to the limited body of research on the health of pastoralist communities living in the horn of Africa, of which there are an estimated 20-30 million [26,27].

The present study found a medium prevalence of IPIs (35%) of which 30.6% were single infections and 4.4% were a co-infection. The most prevalent IPI was the protozoan parasite *Giardia lamblia* (21.9%) followed by the helminth parasite *Ascaris lumbricoides* (12.8%). Prevalence of *Entamoeba histolytica/dispar* (3%), Hookworm (1.4%), and *Hymenolepis nana* (0.3%) were all quite low in the present study. A recent study performed in the same region during the dry (drought) season showed slightly higher overall IPI prevalence (47%) than in our study, although the prevalence for *G. lamblia* and *A. lumbricoides* were comparable [25]. Studies in other regions of Ethiopia have also found a medium-high prevalence (between 17% and 58%) of IPIs in pre-school aged children, although most were conducted in communities and ecologies dissimilar to those in the present study [6,11–17].

Bivariate and multivariate analysis of risk factors associated with *Giardia lamblia* infection revealed significant associations with source of drinking water, ownership of cattle, and ownership of chicken. Sourcing from river water and rainwater both showed a high odds of *G. lamblia* infection compared to sourcing from a borehole/spring/tank-truck, indicating a potential contamination of river and rainwater. As described in the results, there is a clear separation of where pastoralists and agro-pastoralists source their water; agro-pastoralists source mainly from the Shebelle River, while pastoralists source mainly from rainwater collected in open shallow wells (birkads) or from surface water in ditches/pools near roads. Both water sources (river or rain), in comparison with water coming from a borehole, natural

spring, or tank truck, represent much higher chances of environmental and animal/human contamination. These sources are especially at risk of contamination during rainy seasons, when flooding is common and soils contaminated with human and animal feces, due to high levels of open defecation, are carried into nearby rivers and streams as well as open wells/ditches. Other studies have demonstrated that poor water quality or feces contaminated water contributes to *G. lamblia* (and other parasites) transmission and prevalence in humans and animals [6,7,38–40]. In addition, water security in the region is a recurrent problem, with drought-intensity increasing every year due to climate change [41,42]. Therefore, in combination with drought-related water shortages and contamination of important water sources, the water security situation in this region for pastoralists may worsen and impact human and animal health even more [43]. Our results therefore represent a strong indication for priority to be given to constructing and maintaining improved water sources for agro-pastoralists and pastoralists in the Adadle woreda and wider ESRS.

Ownership of cattle was also found to be significantly associated with *Giardia lamblia* infection in bivariate and multivariate analyses. With increasing numbers of cattle, categorically evaluated, the odds of infection also increased. A similar effect was seen for ownership of chicken, although this result is limited by the small number of participants living in households owning chicken (N=18) and the fact that no pastoralist participants owned chicken. The association of *G. lamblia* with chicken ownership is therefore more applicable to agro-pastoralist communities. Our results are supported by the fact that *G. lamblia* is a zoonotic parasite, with the ability to infect and be transmitted to and from many mammals, including cattle, chicken, dogs, cats, and other livestock [7,44–47]. Transmission of *G. lamblia* from cows or chicken to children could therefore occur through several direct and indirect pathways; children playing around household animals may become exposed to animal feces containing

cysts, eggs, or larva, through touching or inhalation; and animals and humans defecating in similar spots may allow for cross-over contamination if proper hygiene measures are not observed [48–51]. Additionally, in the present study, humans and animals from pastoralist communities have shared water sources and may also defecate in or around water sources. Therefore, water presents a highly probable place for transmission between species, indicating that a One Health approach to tackling *Giardia lamblia* in pastoralist communities is warranted [39]. In addition, a recent study showed high levels of animal syndromes in pastoralist livestock in Adadle, with cattle and sheep showing high rates of gastrointestinal diseases [28,52]. Due to the close human-animal relationship in pastoralist communities, there may be shared risk factors for gastrointestinal diseases in humans and animals in pastoralist communities [28,52]. Therefore, improved access to safe water and proper sanitation for (agro-) pastoralist communities in Adadle would likely not only improve the health of humans, especially children, but also the animals they rely on for food and livelihood.

Bivariate analysis revealed children aged 3 – 4 years old were positively associated with *G. lamblia* infection. Similarly, multivariate analysis for *A. lumbricoides* infection revealed a significant positive association with the age group 3 – 4 years, in comparison to 2 – 3 years and 4 – 5 years. As both *A. lumbricoides* and *G. lamblia* are transmitted via the faecal-oral route [2,6,39], it is possible that this age group exhibits a specific behavioural trait that increases their risk of acquiring these parasites. Indeed, a study on children aged 6 - 60 months in the Southern Ethiopian region found the peak in infection for both *A. lumbricoides* and *G. lamblia* infection was for the 3 - 4 year age group [16]. However, several other studies on the same parasites have found peaks in younger or in older age groups [17,53], indicating that the specific social and ecological context likely plays a role. Indeed, age-related changes in behaviour around play and eating, increased ability and independence to move around, loss of

immunologic protection from breastmilk following the weaning period, and other developmental changes throughout childhood are all potential reasons for increased risk of IPIs and may vary in different cultural contexts [16,53]. Further qualitative and ethnographic studies are warranted to elucidate the specific factors leading to this result.

The prevalence of wasting 30%, stunting 14%, underweight 17% and low MUAC 2.3% measured in our study group reflect the findings of a similar study performed during the dry season in 2019 (wasting 34%, stunting 30%, underweight 40%, low MUAC 15.9%), although the prevalences we found are notably lower, especially for low MUAC, which is another indicator of wasting or acute malnutrition [25]. The lower prevalence for wasting, stunting, underweight and low MUAC compared to the previous study may be due seasonal variations in nutritional and health status of (agro-)pastoralist children; during the wet season, when our study took place, the economic and nutritional status of pastoralist communities improves, due to more food availability and healthier animals [25,54]. This effect of seasonality is also expected to be true for parasitic infections [38,43]. The low concordance between wasting status (30%) and low MUAC (2.3%), both indicators of acute undernutrition or wasting [55], is a common occurrence that has been noted by several studies, likely due to the difference in what illness profile each measure captures, as well as the non-specific cut-offs for MUAC, irrespective of the child's age or sex [56–59].

Multivariate analyses revealed male children in our study had a higher odds of being wasted, stunted, and underweight, which reflects other findings in Ethiopia and Sub-Saharan Africa [60,61], but which remain poorly understood socially and physiologically. In addition, wasting was positively associated with children aged 3 – 4 and 4 – 5 years old, and negatively associated with *G. lamblia* infection. The former result has been reflected in other studies of under 5 children in this region of Africa [60,62] and is possibly due to age-related changes in diet and hygiene behaviours. The latter result is more surprising, as it contradicts other studies that

found positive associations between *G. lamblia* or IPIs in general with wasting status [25,53]. This may be due to the seasonal variations in wasting and *G. lamblia* prevalence/transmission, respectively, muddling the true relationship between them. Finally, underweight was also negatively associated with pastoralist children, which may be due to socioeconomic differences between pastoralist and agro-pastoralist communities, such as differences in animal ownership, food consumption, income and environmental risk factors. These potential differences and their impact on health should be assessed in more detail in future anthropological-epidemiological studies.

There are three limiting factors of the presents study due to logistical constraints. First, due to the methods used (inclusion and exclusion criterion) in selecting children for this study, it is possible that we overestimated wasting and stunting in the population and underestimated IPIs; children receiving antibiotics for any type of illness (which could include symptomatic IPIs) were excluded from the study. Further, only one sample was obtained and analysed per child, which may underestimate the true prevalence of IPIs, since the diagnostic techniques used lack perfect sensitivity for certain intestinal parasites [32,63]. Given the strong associations of water and animal exposures to *G. lamblia* infection risk in this study, future studies should include environmental and animal parasitology when assessing parasite prevalence and risk factors in communities in which there is a high rate of contact between humans, animals and their environment, as is the case in the given study population. In addition, a One Health approach in parasitology and pathogenic microorganism research is applicable beyond the few parasites descriptively and statistically analysed in this study; indeed, there are many parasites and microorganisms of human and animal health importance that are transmitted among and between humans and animals through environmental channels (water, soil, food, plants, air) [6–8,38,39,64]. Transmission events between humans, animals, and their environment would

best be addressed using genomic techniques [65], and supported with qualitative information to place the transmission events in context and develop appropriate interventions in the given context [66,67]. Taking these approaches would likely improve the health of humans and their animals, as well as reduce the loss of food and income to illness [8,10].

Conclusions

The high prevalence of IPIs in young agro-pastoralist and pastoralist children (aged 2 - 5), especially high prevalence of *G. lamblia*, are of regional public health concern, given the immediate and long-term health impacts of infection in children. Drinking water source and contact with cattle and chickens were found to be important risk factors for *G. lamblia* infection in these communities. Additionally, the high prevalence of wasting, stunting, and underweight, even during the wet season, are indicators that the food security in the region is still inadequate. We recommend additions and improvements to water and sanitation infrastructure for use by semi-mobile pastoralists, as well as nutritional and health interventions aimed at improving the nutritional status and health of children. These interventions would undoubtedly also improve the health of pastoralist animals and should be paired with increased surveillance and veterinary treatment of pastoralist animals. Any intervention should be implemented in a transdisciplinary manner and seek to involve actors from governmental agencies for humans, animals, and the environment, for the improved health of small children in pastoralist communities.

Key Words

Ethiopian Somali Regional State, Ethiopia, children <5 years, intestinal parasitic infection, *Giardia lamblia*, *Ascaris lumbricoides*, pastoralist, One Health, WASH, livestock animals

Abbreviations

IPIs, intestinal parasitic infections; ESRS; Ethiopian Somali Regional State; WASH; water, sanitation, and hygiene; WHO, World Health Organisation; MUAC, mid-upper arm circumference; EBF, exclusive breastfeeding.

Data Availability

All data can be found in the Supplementary Material of this article.

Funding

This study received financial support from the Swiss Agency for Development and Cooperation (SDC) (AM, no. 7F-09057.01.02), the Nutricia Research Foundation (PV, no. 2019-20), the Forschungsfonds of the University of Basel (PV, Fellowship in 2020), the SNSF Return Grant (PV, no. P3P3PA_177877) and the Eccellenza Fellowship (PV, no. PCEFP3_194545). The funders had no role in study design, data collection and analysis, decision to publish, or preparation of the manuscript.

Conflicts of Interest

The authors declare that they have no conflict of interest.

Contributions

AM, PV, RT, JH and CS and JZ designed the study; AM, RT, and PV managed study sites and sample collection; AM, RBY and SBH collected and processed biological samples; KL

analysed study data with guidance from JH and AM; KL wrote the first draft; and all the authors reviewed and approved the final manuscript.

OrcID

Abdifatah Muktar Muhummed https://orcid.org/0000-0002-1966-729X

Pascale Vonaesch https://orcid.org/0000-0002-1064-6505

Jakob Zinsstag https://orcid.org/0000-0002-8899-6097

Jan Hattendorf https://orcid.org/0000-0002-1176-401X

Supporting information

S1 Fig. Methodological and analytical flow of study.

S2 Fig. Household WASH characteristics of agro-pastoralist and pastoralist children aged 2 - 5 years living in Adadle woreda, Somali region, Ethiopia. Alternative informational figure with Somali translations.

S1 Table. Multivariate analysis of wasting status of agro-pastoralist and pastoralist children aged 2 to 5 years living in Adadle woreda, Somali region, Ethiopia.

S2 Table. Multivariate analysis of stunting status of agro-pastoralist and pastoralist children aged 2 to 5 years living in Adadle woreda, Somali region, Ethiopia.

S3 Table. Multivariate analysis of underweight status of agro-pastoralist and pastoralist children aged 2 to 5 years living in Adadle woreda, Somali region, Ethiopia.

S4 Table. Sociodemographic household characteristics of agro-pastoralist and pastoralist children aged 2 - 5 years living in Adadle woreda, Somali region, Ethiopia.

S5 Table. Household animal herds of agro-pastoralist and pastoralist children aged 2 - 5 years of age living in Adadle woreda, Somali region, Ethiopia.

Acknowledgments

We would like to thank the agro-pastoralist and pastoralist communities in this study and especially the mothers of the children who allowed their children to participate. Many thanks go to local guides in finding the pastoralist camps, the data collectors, and the laboratory staff at Jigjiga University: Fuad, Ahmed, Dek, Mohammed Hussien, Mohammed Askari, Mohamoud, and countless others. Additional thanks to the Jigjiga One Health Initiative team at Jigjiga University and the Armauer Hansen Research Institute in Ethiopia and the Human and the Animal Health Unit team at Swiss Tropical Public Health Institute in Switzerland.

References

1. Chifunda K, Kelly P. Parasitic infections of the gut in children. Paediatr Int Child Health. 2019 Feb;39(1):65–72.

2. Prevention and control of intestinal parasitic infections: WHO Technical Report Series N°749 [Internet]. [cited 2022 Jun 16]. Available from: https://www.who.int/publications-detail-redirect/WHO-TRS-749

3. Shaima SN, Das SK, Ahmed S, Jahan Y, Khan SH, Mamun GMdS, et al. Anthropometric Indices of Giardia-Infected Under-Five Children Presenting with Moderate-to-Severe Diarrhea and Their Healthy Community Controls: Data from the Global Enteric Multicenter Study. Children (Basel). 2021 Dec 15;8(12):1186.

4. Magana-Arachchi DN, Wanigatunge RP. Ubiquitous waterborne pathogens. Waterborne Pathogens. 2020;15–42.

5. WHO. Soil-transmitted helminth infections [Internet]. 2020 [cited 2020 Nov 23]. Available from: https://www.who.int/news-room/fact-sheets/detail/soil-transmitted-helminth-infections

6. Gizaw Z, Yalew AW, Bitew BD, Lee J, Bisesi M. Fecal indicator bacteria along multiple environmental exposure pathways (water, food, and soil) and intestinal parasites among children in the rural northwest Ethiopia. BMC Gastroenterol. 2022 Feb 27;22(1):84.

7. Barnes AN, Davaasuren A, Baasandavga U, Lantos PM, Gonchigoo B, Gray GC. Zoonotic enteric parasites in Mongolian people, animals, and the environment: Using One Health to address shared pathogens. PLoS Negl Trop Dis. 2021 Jul 8;15(7):e0009543.

8. Zinsstag J, Schelling E, Crump L, Whittaker M, Tanner M, Stephen C, editors. One Health: The Theory and Practice of Integrated Health Approaches [Internet]. 2nd ed. 2020 [cited 2021 Aug 31]. 464 p. Available from: https://www.cabi.org/bookshop/book/9781789242577/

9. Zinsstag J, Mackenzie JS, Jeggo M, Heymann DL, Patz JA, Daszak P. Mainstreaming One Health. Ecohealth. 2012;9(2):107–10.

10. Zinsstag J, Schelling E, Waltner-Toews D, Tanner M. From “one medicine” to “one health” and systemic approaches to health and well-being. Prev Vet Med. 2011 Sep 1;101(3–4):148–56.

11. Mekonnen HS, Ekubagewargies DT. Prevalence and factors associated with intestinal parasites among under-five children attending Woreta Health Center, Northwest Ethiopia. BMC Infect Dis. 2019 Mar 13;19(1):256.

12. Tsegaye B, Yoseph A, Beyene H. Prevalence and factors associated with intestinal parasites among children of age 6 to 59 months in, Boricha district, South Ethiopia, in 2018. BMC Pediatr. 2020 Jan 22;20(1):28.

13. Zemene T, Shiferaw MB. Prevalence of intestinal parasitic infections in children under the age of 5 years attending the Debre Birhan referral hospital, North Shoa, Ethiopia. BMC Res Notes. 2018 Jan 22;11:58.

14. Asfaw ST, Giotom L. Malnutrition and enteric parasitoses among under-five children in Aynalem Village, Tigray. Ethiopian Journal of Health Development. 2000;14(1):67–75.

15. Wasihun AG, Teferi M, Negash L, Marugán J, Yemane D, McGuigan KG, et al. Intestinal parasitosis, anaemia and risk factors among pre-school children in Tigray region, northern Ethiopia. BMC Infect Dis. 2020 May 27;20(1):379.

16. Mulatu G, Zeynudin A, Zemene E, Debalke S, Beyene G. Intestinal parasitic infections among children under five years of age presenting with diarrhoeal diseases to two public health facilities in Hawassa, South Ethiopia. Infect Dis Poverty. 2015 Nov 4;4:49.

17. Gadisa E, Jote K. Prevalence and factors associated with intestinal parasitic infection among under-five children in and around Haro Dumal Town, Bale Zone, Ethiopia. BMC Pediatr. 2019 Oct 27;19:385.

18. Abdi M, Nibret E, Munshea A. Prevalence of intestinal helminthic infections and malnutrition among schoolchildren of the Zegie Peninsula, northwestern Ethiopia. Journal of Infection and Public Health. 2017 Jan 1;10(1):84–92.

19. Abera A, Nibret E. Prevalence of gastrointestinal helminthic infections and associated risk factors among schoolchildren in Tilili town, northwest Ethiopia. Asian Pacific Journal of Tropical Medicine. 2014 Jul 1;7(7):525–30.

20. Alemayehu B, Tomass Z, Wadilo F, Leja D, Liang S, Erko B. Epidemiology of intestinal helminthiasis among school children with emphasis on Schistosoma mansoni infection in Wolaita zone, Southern Ethiopia. BMC Public Health. 2017 Jun 20;17(1):587.

21. Mahmud MA, Spigt M, Mulugeta Bezabih A, López Pavon I, Dinant GJ, Blanco Velasco R. Risk factors for intestinal parasitosis, anaemia, and malnutrition among school children in Ethiopia. Pathogens and Global Health. 2013 Mar 1;107(2):58–65.

22. Nguyen NL, Gelaye B, Aboset N, Kumie A, Williams MA, Berhane Y. Intestinal Parasitic Infection and Nutritional Status among School Children in Angolela, Ethiopia. J Prev Med Hyg. 2012 Sep;53(3):157–64.

23. Yami A, Mamo Y, Kebede S. Prevalence and predictors of intestinal helminthiasis among school children in jimma zone; a cross-sectional study. Ethiop J Health Sci. 2011 Nov;21(3):167–74.

24. Chelkeba L, Mekonnen Z, Alemu Y, Emana D. Epidemiology of intestinal parasitic infections in preschool and school-aged Ethiopian children: a systematic review and meta-analysis. BMC Public Health. 2020 Jan 28;20(1):117.

25. Osman KA, Zinsstag J, Tschopp R, Schelling E, Hattendorf J, Umer A, et al. Nutritional status and intestinal parasites among young children from pastoralist communities of the Ethiopian Somali region. Matern Child Nutr. 2020 Jul;16(3):e12955.

26. Horn of Africa - Multi-Sectoral Interventions in Pastoralist Communities Fact Sheet #2, Fiscal Year (FY) 2005 - Djibouti | ReliefWeb [Internet]. [cited 2022 Jun 16]. Available from: https://reliefweb.int/report/djibouti/horn-africa-multi-sectoral-interventions-pastoralist-communities-fact-sheet-2-fiscal

27. Mkutu K. Pastoralism and Conflict in the Horn of Africa. 2001;45.

28. Osman Y, Ali SM, Schelling E, Tschopp R, Hattendorf J, Muhumed A, et al. Integrated community based human and animal syndromic surveillance in Adadle district of the Somali region of Ethiopia. One Health. 2021 Dec;13:100334.

29. Myatt M, Guevarra E. zscorer: Anthropometric z-score Calculator [Internet]. nutriverse; 2019 [cited 2022 May 21]. Available from: https://nutriverse.io/zscorer/

30. Hartung C, Lerer A, Anokwa Y, Tseng C, Brunette W, Borriello G. Open Data Kit: Tools to Build Information Services for Developing Regions. In: Proceedings of the 4th ACM/IEEE International Conference on Information and Communication Technologies and Development [Internet]. New York, NY, USA: Association for Computing Machinery; 2010. (ICTD '10). Available from: https://doi.org/10.1145/2369220.2369236

31. Katz N, Chaves A, Pellegrino J. A simple device for quantitative stool thick-smear technique in Schistosomiasis mansoni. Rev Inst Med Trop Sao Paulo. 1972 Dec;14(6):397–400.

32. Utzinger J, Botero-Kleiven S, Castelli F, Chiodini PL, Edwards H, Köhler N, et al. Microscopic diagnosis of sodium acetate-acetic acid-formalin-fixed stool samples for helminths and intestinal protozoa: a comparison among European reference laboratories. Clinical Microbiology and Infection. 2010 Mar 1;16(3):267–73.

33. R Core Team. R: A language and environment for statistical computing. [Internet]. Vienna, Austria: R Foundation for Statistical Computing; 2013. Available from: http://www.R-project.org/

34. Rich B. table1: Tables of Descriptive Statistics in HTML. [Internet]. 2021. Available from: https://CRAN.R-project.org/package=table1

35. Højsgaard S, Halekoh U, Yan J. The R Package geepack for Generalized Estimating Equations. Journal of Statistical Software. 2006;15:1–11.

36. QGIS.org. QGIS Geographic Information System [Internet]. QGIS Association; 2022. Available from: http://www.qgis.org

37. Ethiopia - Subnational Administrative Boundaries - Humanitarian Data Exchange [Internet]. 2021 [cited 2022 Jun 16]. Available from: https://data.humdata.org/dataset/cod-ab-eth

38. Pawestri AR, Thima K, Leetachewa S, Maneekan P, Deesitthivech O, Pinna C, et al. Seasonal prevalence, risk factors, and One Health intervention for prevention of intestinal parasitic infection in underprivileged communities on the Thai-Myanmar border. International Journal of Infectious Diseases. 2021 Apr 1;105:152–60.

39. Lee SC, Ngui R, Tan TK, Roslan MA, Ithoi I, Mahdy MAK, et al. Understanding Giardia infections among rural communities using the one health approach. Acta Tropica. 2017 Dec 1;176:349–54.

40. Archer J, O'Halloran L, Al-Shehri H, Summers S, Bhattacharyya T, Kabaterine NB, et al. Intestinal Schistosomiasis and Giardiasis Co-Infection in Sub-Saharan Africa: Can a One Health Approach Improve Control of Each Waterborne Parasite Simultaneously? Trop Med Infect Dis. 2020 Aug 25;5(3):E137.

41. Osima S, Indasi VS, Zaroug M, Endris HS, Gudoshava M, Misiani HO, et al. Projected climate over the Greater Horn of Africa under 1.5 °C and 2 °C global warming. Environ Res Lett. 2018 May;13(6):065004.

42. Krampe F, Goor L van de, Barnhoorn A, Smith E, Smith D. Water Security and Governance in the Horn of Africa [Internet]. SIPRI; 2020 Mar [cited 2022 Jun 16]. Available from: https://www.sipri.org/publications/2020/sipri-policy-papers/water-security-and-governance-horn-africa

43. VanderWaal K, Gilbertson M, Okanga S, Allan BF, Craft ME. Seasonality and pathogen transmission in pastoral cattle contact networks. R Soc Open Sci. 2017 Dec 6;4(12):170808.

44. Kuthyar S, Kowalewski MM, Seabolt M, Roellig DM, Gillespie TR. Molecular characterization of Giardia duodenalis and evidence for cross-species transmission in Northern Argentina. Transboundary and Emerging Diseases [Internet]. 2021 Jul [cited 2022 Apr 21];n/a(n/a). Available from: https://onlinelibrary.wiley.com/doi/abs/10.1111/tbed.14220

45. Sack A, Palanisamy G, Manuel M, Paulsamy C, Rose A, Kaliappan SP, et al. A One Health Approach to Defining Animal and Human Helminth Exposure Risks in a Tribal Village in Southern India. Am J Trop Med Hyg. 2021 May 24;105(1):196–203.

46. Barnes AN, Davaasuren A, Baasandagva U, Gray GC. A systematic review of zoonotic enteric parasitic diseases among nomadic and pastoral people. PLoS One. 2017;12(11):e0188809.

47. Thompson RCA. The zoonotic significance and molecular epidemiology of Giardia and giardiasis. Veterinary Parasitology. 2004 Dec 9;126(1):15–35.

48. Zambrano LD, Levy K, Menezes NP, Freeman MC. Human diarrhea infections associated with domestic animal husbandry: a systematic review and meta-analysis. Trans R Soc Trop Med Hyg. 2014 Jun;108(6):313–25.

49. Delahoy MJ, Wodnik B, McAliley L, Penakalapati G, Swarthout J, Freeman MC, et al. Pathogens transmitted in animal feces in low- and middle-income countries. Int J Hyg Environ Health. 2018 May;221(4):661–76.

50. Penakalapati G, Swarthout J, Delahoy MJ, McAliley L, Wodnik B, Levy K, et al. Exposure to Animal Feces and Human Health: A Systematic Review and Proposed Research Priorities. Environ Sci Technol. 2017 Oct 17;51(20):11537–52.

51. Balderrama-Carmona AP, Gortáres-Moroyoqui P, Álvarez-Valencia LH, Castro-Espinoza L, Mondaca-Fernández I, Balderas-Cortés J de J, et al. Occurrence and quantitative microbial risk assessment of Cryptosporidium and Giardia in soil and air samples. Int J Infect Dis. 2014 Sep;26:123–7.

52. Bauhofer AFL, Cossa-Moiane I, Marques S, Guimarães EL, Munlela B, Anapakala E, et al. Intestinal protozoan infections among children 0-168 months with diarrhea in Mozambique: June 2014 - January 2018. PLoS Negl Trop Dis. 2020 Apr 22;14(4):e0008195.

53. Bechir M, Schelling E, Hamit MA, Tanner M, Zinsstag J. Parasitic infections, anemia and malnutrition among rural settled and mobile pastoralist mothers and their children in Chad. Ecohealth. 2012 Jun;9(2):122–31.

54. Sadler K, Mitchard E, Abdi A, Shiferaw Y, Bekele G, Catley A. MILK MATTERS The Impact of Dry Season Livestock. :52.

55. WHO and UNICEF. WHO child growth standards and the identification of severe acute malnutrition in infants and children [Internet]. 2009 [cited 2022 Jul 7]. Available from: https://www.academia.edu/11921202/WHO_child_growth_standards_and_the_identification_of_severe_acute_malnutrition_in_infants_and_children

56. Tadesse AW, Tadesse E, Berhane Y, Ekström EC. Comparison of Mid-Upper Arm Circumference and Weight-for-Height to Diagnose Severe Acute Malnutrition: A Study in Southern Ethiopia. Nutrients. 2017 Mar 11;9(3):267.

57. Berkley J, Mwangi I, Griffiths K, Ahmed I, Mithwani S, English M, et al. Assessment of severe malnutrition among hospitalized children in rural Kenya: comparison of weight for height and mid upper arm circumference. JAMA. 2005 Aug 3;294(5):591–7.

58. Laillou A, Prak S, de Groot R, Whitney S, Conkle J, Horton L, et al. Optimal Screening of Children with Acute Malnutrition Requires a Change in Current WHO Guidelines as MUAC and WHZ Identify Different Patient Groups. PLoS One. 2014 Jul 1;9(7):e101159.

59. Bilukha O, Leidman E. Concordance between the estimates of wasting measured by weight-for-height and by mid-upper arm circumference for classification of severity of nutrition crisis: analysis of population-representative surveys from humanitarian settings. BMC Nutrition. 2018 May 18;4(1):24.

60. Akombi BJ, Agho KE, Hall JJ, Wali N, Renzaho AMN, Merom D. Stunting, Wasting and Underweight in Sub-Saharan Africa: A Systematic Review. Int J Environ Res Public Health. 2017 Aug;14(8):863.

61. Wamani H, Åstrøm AN, Peterson S, Tumwine JK, Tylleskär T. Boys are more stunted than girls in Sub-Saharan Africa: a meta-analysis of 16 demographic and health surveys. BMC Pediatr. 2007 Dec;7(1):1–10.

62. Agho KE, Akombi BJ, Ferdous AJ, Mbugua I, Kamara JK. Childhood undernutrition in three disadvantaged East African Districts: a multinomial analysis. BMC Pediatr. 2019 Dec;19(1):1–11.

63. Stensvold CR, Nielsen HV. Comparison of Microscopy and PCR for Detection of Intestinal Parasites in Danish Patients Supports an Incentive for Molecular Screening Platforms. Journal of Clinical Microbiology. 2012 Feb;50(2):540–1.

64. CDC. Zoonotic Diseases | One Health | CDC [Internet]. 2021 [cited 2021 Aug 31]. Available from: https://www.cdc.gov/onehealth/basics/zoonotic-diseases.html

65. Angora EK, Vangraefschepe A, Allienne JF, Menan H, Coulibaly JT, Meïté A, et al. Population genetic structure of Schistosoma haematobium and Schistosoma haematobium × Schistosoma bovis hybrids among school-aged children in Côte d'Ivoire. Parasite. 2022;29:23.

66. Hughes SD, Woods WJ, O'Keefe KJ, Delgado V, Pipkin S, Scheer S, et al. Integrating Phylogenetic Biomarker Data and Qualitative Approaches: An Example of HIV Transmission Clusters as a Sampling Frame for Semistructured Interviews and Implications for the COVID-19 Era. Journal of Mixed Methods Research. 2021 Jul 1;15(3):327–47.

67. Kaur M. Application of Mixed Method Approach in Public Health Research. Indian J Community Med. 2016;41(2):93–7.

Figure 1

Click here to access/download;Figure;Figure 1.png

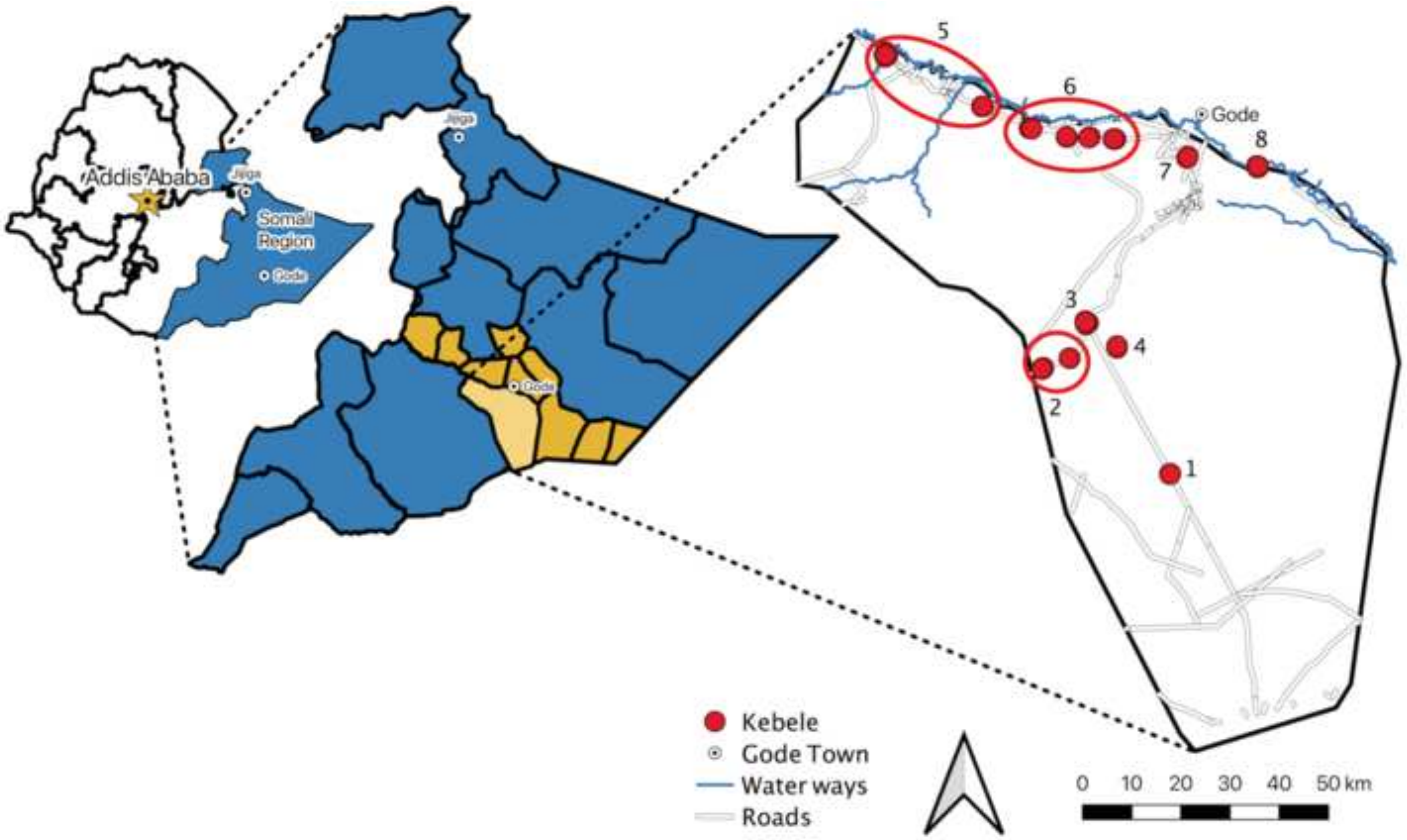

Figure 2

Click here to access/download;Figure;Figure 2.png

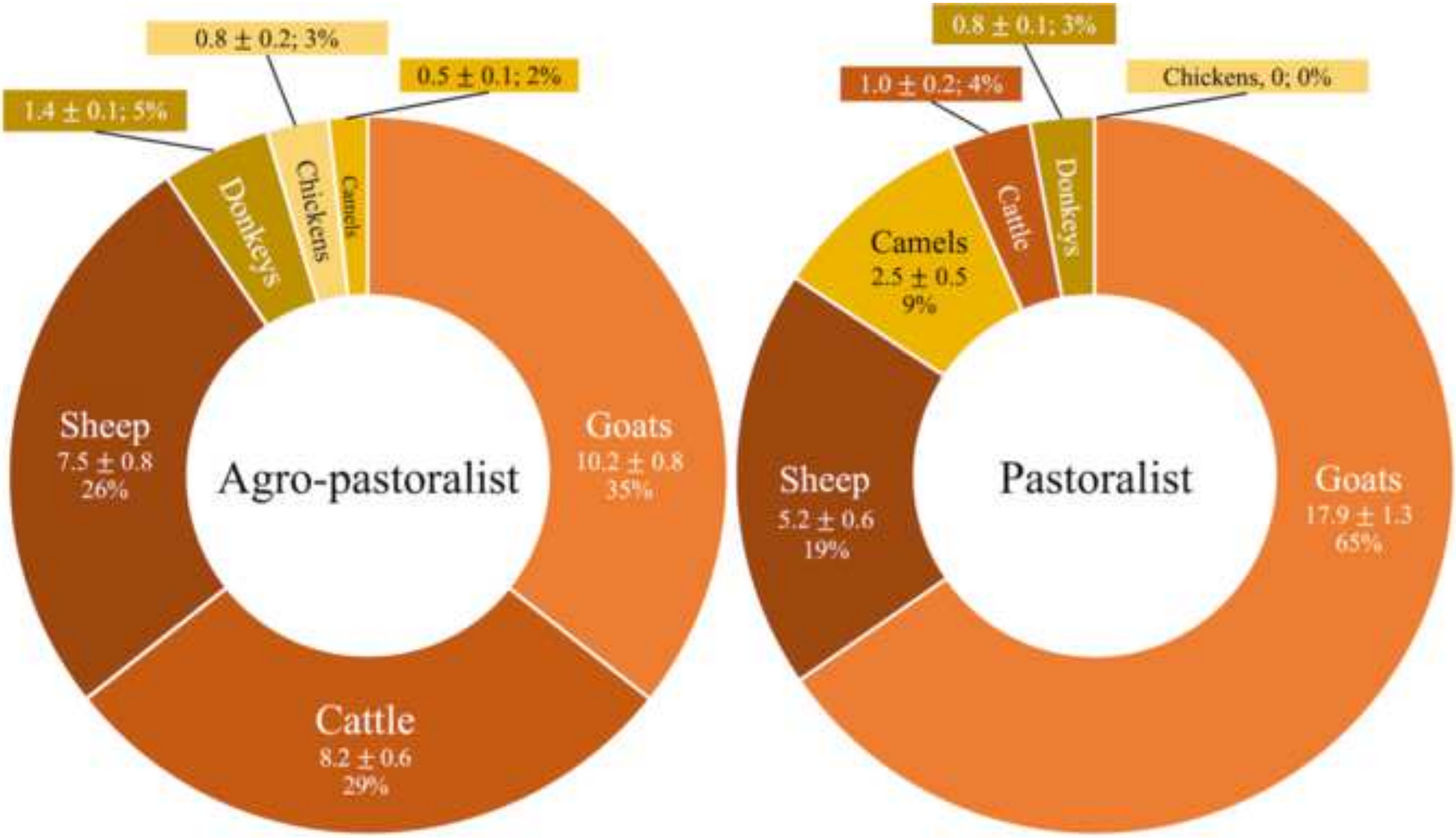

Figure S1

Click here to access/download
Supporting Information
S1_fig.pdf

Figure S2

Click here to access/download
Supporting Information
S2_fig.pdf

Table S1

Click here to access/download
Supporting Information
S1_table.docx

Table S2

Click here to access/download
Supporting Information
S2_table.docx

Table S3

Click here to access/download
Supporting Information
S3_table.docx

TTable S4

Click here to access/download
Supporting Information
S4_table.docx

Table S5

Click here to access/download
Supporting Information
S5_table.docx
