# Supplementary material for: Prevalence and associated risk factors of intestinal parasitic infections among children in pastoralist and agro-pastoralist communities in the Adadle woreda of the Somali Regional State of Ethiopia
Source: PLoS Negl Trop Dis. 2023 Jul 3;17(7):e0011448. doi: 10.1371/journal.pntd.0011448 (PMC10348586; doi:10.1371/journal.pntd.0011448)
Supplement: Attachment — Submitted filename: ResponseLetterReviewersPlosNTD_250523.pdf [file pntd.0011448.s013.pdf]

UNIL | Université de Lausanne
Département de microbiologie fondamentale
bâtiment Biophore bureau BIO-2310.1::02
CH-1015 Lausanne

Prof. Pascale Vonaesch
Department of Fundamental Microbiology
University of Lausanne
Campus UNIL-Sorge
1015 Lausanne

Lausanne, the 25th of May 2023

Subject: Point-to-point reply for the second revisions regarding the manuscript in Plos NTD

Dear editor, dear reviewers

We would like to thank the reviewers for their second review, comments and criticisms of the revised manuscript entitled "**Prevalence and associated risk factors of intestinal parasitic infections among children in pastoralist and agro-pastoralist communities in the Adadle woreda of the Somali Region of Ethiopia**", which further improved the quality of the manuscript.

In the point-to-point reply provided below, we include our answers to the points raised by the reviewers and describe the overall changes made on the revised manuscript. We also submit the new version of the manuscript including tracked changes, a clean version as well as the revised supplement.

We hope the manuscript in its revised form is now acceptable for publication in Plos NTD.

Yours sincerely,

Pascale Vonaesch and co-authors

Tél.+41 21 692 56 09 | Fax.+41 21 692 56 05 | pascale.vonaesch@unil.ch

Point-to-point reply

Methods

Reviewer #1: None

Reviewer #2: (No Response)

Reviewer #3: The objectives are clearly stated and the study designed appropriately addressed the objectives. The population is clearly defined but the expected prevalence used to calculate the sample size is inappropriate. The sample size is adequate and no concern about the ethical requirement.
Thank you for your feedback. We have rewritten the sentence for clarity, lines (136-137): "A previous study in children aged 2 to 5 years in this region showed a prevalence of intestinal parasitic infections of 42% [25]."

Results

Reviewer #1: None

Reviewer #2: (No Response)

Reviewer #3: The result is completely presented and analysed with appropriate tool. The figures and tables are adequate and clearly presented
Thank you.

Conclusions

Reviewer #1: None

Reviewer #2: (No Response)

Reviewer #3: The conclusion support the data presented and limitation well spelt out
Thank you.

Editorial and Data Presentation Modifications

Reviewer #1: None

Reviewer #2: (No Response)

Reviewer #3: (No Response)

Summary and General Comments

Reviewer #1: In the current revised manuscript, the authors have addressed my comments and I am satisfied with the way they have improved the manuscript. However, few minor corrections need to be considered. Thank you.

1. Lines 45-48: Conclusions' statement can be further modified to "Improving access to safe water, sanitation, and hygiene services in Adadle and employing a One Health approach would likely improve the health of children living in (agro-) pastoralist communities in Adadle and ESRS; however, further studies are required.".
   Thank you for this suggestion. It has been incorporated.

2. Line 460: "…. should be considered …."
   Thank you. We have removed part of the sentence, so it makes more sense grammatically.

3. Line 462: "…….. were more likely to be infected with ….". Also, modify the sentence in line 502.
   Thank you for this feedback. As our statistics were odds ratios, we purposefully did not use the word "likely" in this manuscript, as this is easily mistaken with likelihood or probability statistics. We have rephrased this sentence to the following, line (467): "Children aged 36 to 47 months old had a higher odds of being infected with *A. lumbricoides*."

4. Line 475: "There are some limitations in the present study …."
   Thank you. It has been incorporated.

5. Line 475: remove "First".
   Agree and changed.

6. Lines 476-479: this long sentence can be split in order to improve clarity.
   Thank you for your feedback and we have changed the text accordingly.

7. Line 500: "Source of drinking water (rain and river), …"
   Thank you and changed.

8. IPI or IPIs? Please ensure consistency throughout the entire text.
   Thank you for this feedback. IPI was used when the singular form was necessary. However, we have changed the sentence structure in the places where IPI previously was, so that IPIs can be used instead. This has been reflected throughout the manuscript.

9. Lines 39, 203, 341, 342, and Table 4: change to "hookworm (Ancylostoma spp. / Necator americanus)"
   Thank you for this detail, we have incorporated this change throughout the manuscript.

10. Line 347: "……; hookworm 2/16."
    Thank you and changed.

Reviewer #2: (No Response)

Reviewer #3:

1. General comment: The MS is well structured but full of grammatical errors and expressions that need to be corrected and as such the authors may need native English expert. Also, the materials and methods is not explicit especially in the area of parasite identification to the species level. My specific comments areas follow.
   Thank you for your feedback. We had a native English expert go again through the newest resubmitted manuscript to check for grammatical errors. The identification of parasites has been addressed in the manuscript. *Entamoeba* is now referred to on a species level as *Entamoeba* spp., while Hookworm is referred to as hookworm (*Ancylostoma duodenale /Necator americanus*). We also added the following in

the methods section, line(208-212): “Although microscopy can only be used to identify *Giardia* at the genus level, *Giardia intestinalis* is the only species of *Giardia* that has been found to infect humans. Therefore, all *Giardia* identified through microscopy in the human faecal samples are referred to as *Giardia intestinalis* throughout the manuscript."

2. Line 25: Delete young from the young children
Thank you for this suggestion. This was addressed in the resubmitted manuscript and is therefore no longer mentioned in the newest manuscript.

3. Line 34: Re-cast as: Protozoan parasite prevalence was 24.9% and 21.9% for E. hystolitica/dispar and Giardia lambda, respectively.
Thank you for your feedback. We have re-written this sentence for clarity.

4. Line 39-41: The highlighted should be recast for clarity
Thank you and done.

5. Line 50: Delete, in
Thank you and completed.

6. Line 53: Delete the highlighted
Thank you and done.

7. Line 67: Consider deleting the: Young
Thank you for this suggestion. This was addressed in the resubmitted manuscript and is therefore no longer mentioned in the newest manuscript.

8. Line 115-116: The highlighted should read: obtained from the parent
Thank you for this suggestion. The edited sentence has been moved to the “ethics statement” section of the methods, lines (115-116): “Oral or written consent was obtained from the parent of all participating children before study enrolment.”

9. Line 122-128: Include the map of the study area
Thank you for this suggestion. We have included a map (Figure 1) of the study area in the Results section, page 14.

10. Line 133-134: You cannot use expected prevalence to calculate the sample size but already established prevalence in that area or elsewhere.
Thank you for your feedback. We have rewritten the sentence for clarity, lines (136-137): “A previous study in children aged 2 to 5 years in this region showed a prevalence of intestinal parasitic infections of 42% [25].”

11. Line 156-157:If you want to refer to young children, then you must define the age group that constitute the young and the non-young children
Thank you for this suggestion and we agree. This was addressed in the resubmitted manuscript and is therefore no longer mentioned in the newest manuscript.

12. Line 194: You may consider deleting : and aliquoted
Thank you for this suggestion. This was addressed in the resubmitted manuscript and has been rewritten as follows, lines (196-197): “The samples were aliquoted and then immediately transferred to a cold box containing ice.”

13. Line 202: Do you mean : Normal saline?
Thank you and we agree. Done.

14. Line 204: At what magnification
Thank you and changed.

15. Line 206: Delete: stool
Thank you and done.

16. Line 207: Name the antiparasitic agent used.
Thank you for this suggestion. This was addressed in the resubmitted manuscript. You can find the antiparasitic agents used in the "ethics statement" section of the methods, lines (116-118): "If a child was found to have an intestinal parasitic infection, the child's mother was given anti-parasitic medications (mebendazole, albendazole or metronidazole) to administer to her child."

17. Line 222: Delete: (N=366)
Thank you and done.

18. Line 233: Delete; even
Thank you and completed.

19. Line 321: First, indicate the overall prevalence (Single+multiple infection) before stratifying to single, multiple prevalences
Thank you and this has been incorporated throughout the manuscript.

20. Line 325: How did you differentiate to species level? Because you used microscopy
Thank you for this feedback and the identification of parasites has been addressed in the manuscript. *Entamoeba* is now referred to on a species level as *Entamoeba* spp., while Hookworm is referred to as hookworm (*Ancylostoma duodenale /Necator americanus*). We also added the following in the methods section, line(208-212): "Although microscopy can only be used to identify *Giardia* at the genus level, *Giardia intestinalis* is the only species of *Giardia* that has been found to infect humans. Therefore, all *Giardia* identified through microscopy in the human faecal samples are referred to as *Giardia intestinalis* throughout the manuscript."

21. Generally, in the result section, I think the detection of the Giardia and Entamoeba should stop at the genus level. Also, pictures of the detected parasites would add a lot of value to this MS. I also think that you should mention the hookworms encountered in this study instead of lobbing all of them together.
Thank you for this feedback and the identification of parasites has been addressed in the manuscript. *Entamoeba* is now referred to on a species level as *Entamoeba* spp., while Hookworm is referred to as hookworm (*Ancylostoma duodenale /Necator americanus*). We also added the following in the methods section, line(208-212): "Although microscopy can only be used to identify *Giardia* at the genus level, *Giardia intestinalis* is the only species of *Giardia* that has been found to infect humans. Therefore, all *Giardia* identified through microscopy in the human faecal samples are referred to as *Giardia intestinalis* throughout the manuscript." Regarding pictures of detected parasites, we were unable to take pictures in the field during data collection.

22. Line 372-378: The highlighted should come towards the end before concluding the discussion
Thank you and this was addressed in the resubmitted manuscript.

23. Line 380-384:The highlighted are results that you have mentioned in the result section. So, consider deleting and just discuss what could be responsible for the prevalence reported in this study compared to what has been done elsewhere.
    Thank you and this was addressed in the resubmitted manuscript. All repeated results have been removed from the discussion section.

Comments to Authors

1. The authors have addressed most of the comments given for the first manuscript submission.
   Thank you.

2. Minor comments: Please use "WASH" consistently throughout the manuscript. In some places, "WaSH" is used.
   Thank you for this feedback and we have incorporated it throughout the manuscript.

3. **Results**: On page 17, line 351 in Table 4, the frequency is clearly shown for each category under each variable. But, in the bracket corresponding to each frequency, the symbol for percentage (%) is missing. Please put % in each category.
   Thank you. This has now been done in Table 4.

4. **Discussion**: Please delete the adjective "nearby" (line 397: page 21) as Chad is far away from Ethiopia. You can say simply "pastoralists in Chad were found to have a higher prevalence of IPIs (60%) [44].
   Agree and changed.
