# Supplementary material for: Prevalence and associated risk factors of intestinal parasitic infections among children in pastoralist and agro-pastoralist communities in the Adadle woreda of the Somali Regional State of Ethiopia
Source: PLoS Negl Trop Dis. 2023 Jul 3;17(7):e0011448. doi: 10.1371/journal.pntd.0011448 (PMC10348586; doi:10.1371/journal.pntd.0011448)
Supplement: Attachment — Submitted filename: Comments to authors.doc [file pntd.0011448.s009.doc]

Comments to authors

Prevalence and associated risk factors of intestinal parasitic infections in young pastoralist and agro-pastoralist children in the Somali Region of Ethiopia (MS ID: PNTD-D-22-01533)

**General comments**: The manuscript is well written. The study is also well designed. The objectives are also clear and addressed with appropriate parasitological and clinical methods. There are however some minor points to be addressed by authors.

-Some local names (e.g., Kebele, Woreda etc) should be first defined and be italicized to make it clear to readers who do not know about administrative units in Ethiopia.

**Title:**

- The title can be modified by omitting “young”. It is good to say simply “Prevalence and………………. parasitic infections in pastoralist and agro-pastoralist children……………..”

**Abstract**:

_ Although the main focus of the study is about the prevalence of IPIs, it is also good to include the degree of wasting and stunting in the result section of the abstract.

**Introduction**

In the last paragraph (page 5), study participants close interaction with animals (cattle/ goats/ camels) is emphasized. It is also good to describe their living condition and interactions among neighborhood, especially in raising children as this would increase the risk of transmission of IPIs.

**Selection criteria**

In line 151, it is mentioned that stunting and wasting were done according to WHO. Which protocol? Please show the specific year of publication by WHO (see line 151 and 173).

**Stool samples…**

In lines 206 and 207, it is indicated that children who tested positive were given anti-parasitic drugs. It is good to move this statement to “Ethical Consideration” part. Please mention the names of the drugs administered.

**Statistical analysis**

-Although it is common to use “bivariate” and “univariate” analysis interchangeably, it is preferred to use the latter (univariate) as it can be clearly distinguished from multivariate analysis. “Bivariate” looks as if it were you used two variables in the analysis. In logistic regression model build-up, it is recommended to say “ uni- vs-multi”. Use consistently this style throughout the manuscript.

_Nothing is mentioned about cut-off point of p-values for purposeful selection of variables for multivariate logistic regression build-up (please see the article by Bursac et al., 2008; purposeful selection of variables…..)

**Results**

In table 1 and others, the age of the children was classified as “2-3”, “3-4” and “4-5” years. I checked if there was double-counting. But, the numbers (frequency) are correct. It is a bit confusing about the overlap of age categories. So, make it clear by using legend under the first table or using month instead of year for estimation of the age of study participants.

- In table 4, please use the lower case for first letters for specific names of the genus *Entamoeba*
- What do you mean by “positive association” (line 343 and others). In statistics, there is difference between “association” and “correlation.” We say positive/negative for correlation, but not for association. What has been described here shows the association as it relates to your hypothesis/research question. Please avoid the word “positive” throughout the manuscript. I think you want to say “there is significant statistical association between *G. lamblia* infection and the risk factors (e.g., age, source of water, ownership of cattle).
- Please re-write the statements describing the odds of the likelihood of events. “Sourcing drinking water……. had an adjusted odds ratio of……….” In multivariate analysis, you have already done an adjustment, so no need of describing as “ had an adjusted odds ratio etc”. Instead show it as “aOR or ACOR” in bracket with 95 % CI.

**Discussion**

-In line 377, “ ….of which there are an estimated 20-30 million” is NOT clear. 20-30 million? Humans/ cattle? Please qualify this statement.

_ Line 380. IPIs prevalence is medium. Why? Overprotection of children not to play outside on ground ? or What? Please give plausible biological/behavioral reasons taking into account both children and parents behaviors/practices . “Outdoor “open field” defecation was quite common in the study area (99.4% in agro-pastoralist area vs 92.8% in pastoralist area).

-Did you compare with similar conditions in pastoralists in neighboring countries (Somalia, Kenya etc if there are any reports in these countries).

-Higher odds of infection with *A. lumbricoides* in children aged 3-4 years. Why? You said that “specific behavioural trait.” Please elaborate this statement. This age is usually the time at which children start to crawl and go outside. This might expose thems to ova of *Ascaris* in soil. Please use an appropriate citation for elaborating this statement.

_How are stunting and wasting compared with national report (if any in the specific age group or < five-year children)?

**Conclusions**

In line 380, it is shown that prevalence of IPIs to be “**medium**.” But, you here concluded that “**high**” prevalence was observed (line 506). The two statements will not go together. What is your reference (WHO report/ national or….? ) to say “ medium” or “high”?
