# Supplementary material for: Prevalence and associated risk factors of intestinal parasitic infections among children in pastoralist and agro-pastoralist communities in the Adadle woreda of the Somali Regional State of Ethiopia
Source: PLoS Negl Trop Dis. 2023 Jul 3;17(7):e0011448. doi: 10.1371/journal.pntd.0011448 (PMC10348586; doi:10.1371/journal.pntd.0011448)
Supplement: Attachment — Submitted filename: Comments to authors for revision1.doc [file pntd.0011448.s011.doc]

Comments to authors

PNTD-D-22-01533R1

Full Title: Prevalence and associated risk factors of intestinal parasitic infections among children

in pastoralist and agro-pastoralist communities in the Adadle woreda of the Somali

Region of Ethiopia

The authors have addressed most of the comments given for the first manuscript submission.

Minor comments: Please use “WASH” consistently throughout the manuscript. In some places, “WaSH” is used.

**Results**

On page 17, line 351 in Table 4, the frequency is clearly shown for each category under each variable. But, in the bracket corresponding to each frequency, the symbol for percentage (%) is missing. Please put % in each category.

**Discussion**

-Please delete the adjective “nearby” (line 397: page 21) as Chad is far away from Ethiopia. You can say simply “pastoralists in Chad were found to have a higher prevalence of IPIs (60%) [44].
